# Inclusion of *trans*-resveratrol in methylated cyclodextrins: synthesis and solid-state structures

**DOI:** 10.3762/bjoc.10.331

**Published:** 2014-12-29

**Authors:** Lee Trollope, Dyanne L Cruickshank, Terence Noonan, Susan A Bourne, Milena Sorrenti, Laura Catenacci, Mino R Caira

**Affiliations:** 1 Centre for Supramolecular Chemistry Research (CSCR), Department of Chemistry, University of Cape Town, Rondebosch 7701, South Africa; 2 Department of Drug Sciences, University of Pavia, Via Taramelli 12, 27100 Pavia, Italy

**Keywords:** cyclodextrin, inclusion complexes, thermal analysis, *trans*-resveratrol, X-ray structures

## Abstract

The phytoalexin *trans*-resveratrol, 5-[(1*E*)-2-(4-hydroxyphenyl)ethenyl]-1,3-benzenediol, is a well-known, potent antioxidant having a variety of possible biomedical applications. However, its adverse physicochemical properties (low stability, poor aqueous solubility) limit such applications and its inclusion in cyclodextrins (CDs) has potential for addressing these shortcomings. Here, various methods of the attempted synthesis of inclusion complexes between *trans*-resveratrol and three methylated cyclodextrins (permethylated α-CD, permethylated β-CD and 2,6-dimethylated β-CD) are described. Isolation of the corresponding crystalline 1:1 inclusion compounds enabled their full structure determination by X-ray analysis for the first time, revealing a variety of guest inclusion modes and unique supramolecular crystal packing motifs. The three crystalline inclusion complexes were also fully characterized by thermal analysis (hot stage microscopy, thermogravimetric analysis and differential scanning calorimetry). To complement the solid-state data, phase-solubility studies were conducted using a series of CDs (native and variously derivatised) to establish their effect on the aqueous solubility of *trans*-resveratrol and to estimate association constants for complex formation.

## Introduction

**Figure 1:**
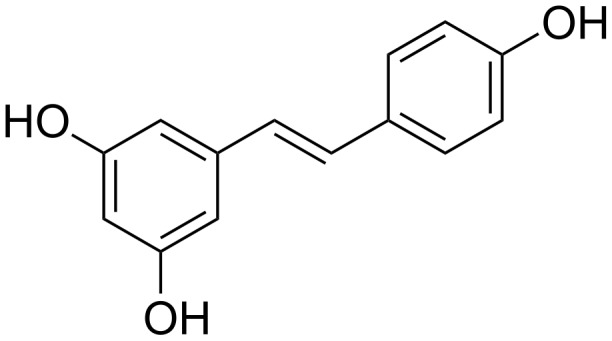
Chemical structure of *trans*-resveratrol (**1**).

The naturally occurring phytoalexin *trans*-resveratrol (5-[(1*E*)-2-(4-hydroxyphenyl)ethenyl]-1,3-benzenediol; *trans*-3,5,4′-trihydroxystilbene, RSV) (**1**, Figure 1), is a triphenolic species which is known to have potent antioxidant activity and consequently a wide range of pharmacological activities [1–2]. In recent years the list of potential medicinal benefits exhibited by RSV (including, e.g., anti-inflammatory effects, cardiovascular protection, and anticancer activity [3]) has increased considerably. Its low aqueous solubility, however, is one of the factors that limits its utility [4] and various methods have been employed to address this shortcoming [5], among them inclusion complexation with cyclodextrins (CDs), which are well-known solubilisers of lipophilic molecules [6].

In addition to enhancing the solubility of guest molecules, CDs can confer chemical stability on bioactive molecules through inclusion of sensitive guest moieties within their hydrophobic cavities [6].

There have been numerous reports of the enhancement in the aqueous solubility of RSV as a result of its inclusion in CDs, most of them based on phase-solubility studies, e.g., [7–8]. In addition, NMR spectroscopic studies have yielded information on solution-state complexation (stoichiometries, association constants) [9]. The latter study was complemented by attempts to characterize putative solid inclusion complexes between CDs and RSV using thermoanalytical, Fourier-transform Infrared (FTIR) spectroscopic and powder X-ray diffraction (PXRD) techniques [9]. In the case of the interaction between α-CD and RSV, for example, the disappearance of the melting endotherm for RSV in the differential scanning calorimetric (DSC) trace of the product was cited as evidence for the formation of a α-CD·RSV complex [9]. The putative inclusion complex between β-CD and RSV, prepared by either the suspension method or using microwave irradiation, yielded highly amorphous products, evident from their PXRD traces [9]; in this case, the absence of characteristic peaks for RSV and the reduction in the degree of crystallinity of the product were considered as indirect proof of complexation. It should be noted that, in general, solid-state characterization using the latter techniques is limited, the evidence for genuine inclusion complex formation not always being definitive because the preparative method may result in one or both components becoming amorphous, or an unexpected solid phase (e.g., a hydrate of the guest compound) might be generated during attempted complexation. Loss of crystallinity of CD inclusion compounds also results when they dehydrate, rendering their PXRD traces less informative.

There is, hitherto, a distinct lack of information on the structural nature of solid-state inclusion complexes between RSV and CDs, despite the fact that such complexes have strong potential for incorporation into tablets or capsules when formulated for medicinal use. A search of the Cambridge Crystallographic Database [10] revealed that no CD·RSV crystal structures have been reported to date.

In this study, various preparative methods were explored in an attempt to generate CD·RSV inclusion complexes with a series of methylated CDs (permethylated α-CD, permethylated β-CD and 2,6-dimethylated β-CD). Here, CD–RSV interaction products were prepared by physical mixing, kneading or co-crystallization from different solutions, by co-evaporation using a rotavapor, or by exposure to microwave radiation. Characterization of the products was achieved using DSC and simultaneous thermogravimetric analysis (TGA/DSC), with support from FTIR spectroscopy and PXRD, where necessary.

An important goal was the isolation of RSV·CD inclusion complexes in crystalline form so that definitive details of the mode of inclusion of the RSV molecule and the packing of complex units could be established by single crystal X-ray analysis. In view of its extended shape and apparent rigidity, the RSV molecule was expected to be partially inserted in the CD host cavities and hence to produce somewhat different supramolecular arrangements in the crystals from those observed with guest molecules having greater conformational freedom. As reported in detail below, the successful isolation of the target inclusion compounds as single crystals enabled their complete structural elucidation, revealing several novel supramolecular features which are relevant for future studies of the antioxidant RSV. Availability of well-defined, crystalline inclusion complexes also ensured that their characterization using thermoanalytical methods could be interpreted on a sound basis. Since a primary application of CDs is enhancement of the solubility of poorly soluble guest molecules [6], phase solubility studies [11] were conducted, and the results, including estimates of complex formation constants, are also reported here.

## Results and Discussion

### Screening for CD–RSV interactions and product characterization

**Figure 2:**
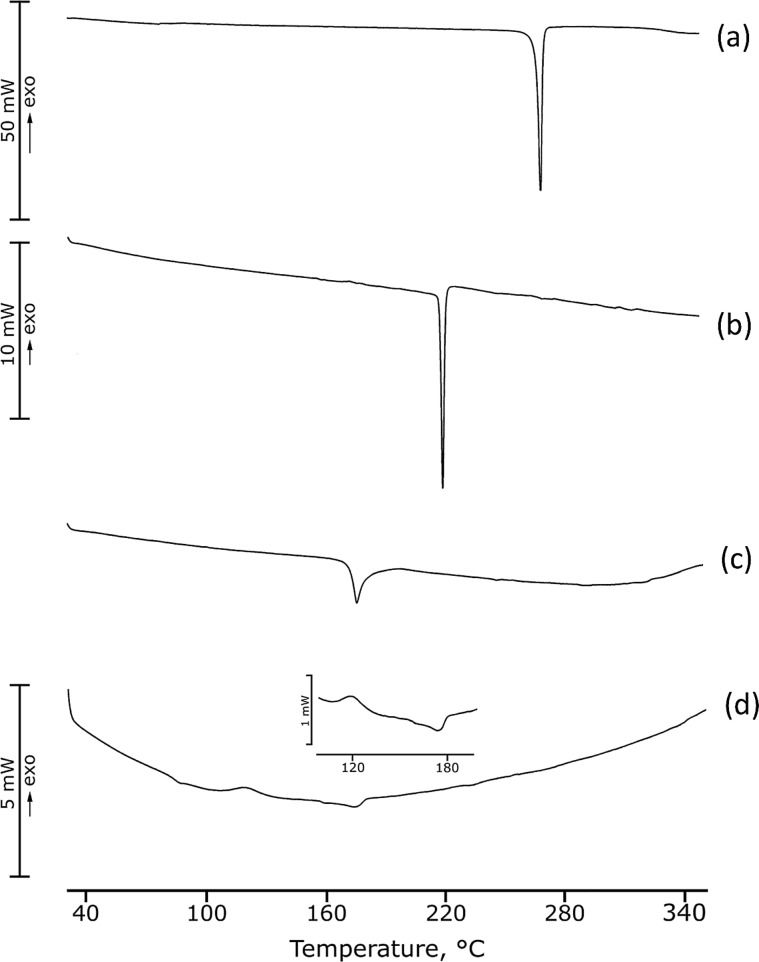
DSC traces of RSV (a), TMA (b), TMA–RSV physical mixture (PM) (c), TMA–RSV preparation by kneading (KN) (d).

Screening for new solid forms of RSV via its interaction with CDs using different preparative methods required preliminary characterization of RSV itself and assessment of the effects on pure RSV of procedures used to prepare the binary systems. Differential scanning calorimetry (DSC) indicated that the commercial product melted at *T*_peak,m_ = 266.3(4) °C (*T*_onset_ = 265.1(3) °C; Δ*H*_m_ = 279(2) J g^−1^) (Figure 2, curve (a)). Thermogravimetric analysis (TGA) revealed mass loss only at 275 °C attributable to sample decomposition (curve not shown). It was ascertained that the kneading treatment (KN) and exposure to irradiation with microwave radiation (MW) had no significant effect on RSV. (Further experimental data on this aspect and all other methods employed in this study are provided in Supporting Information File 1).

On DSC analysis, permethylated α-CD [(hexakis(2,3,6-tri-*O*-methyl)-α-CD; TRIMEA; TMA] yielded an endotherm of fusion only (*T*_peak,m_ = 217.6(1) °C, Δ*H*_m_ = 40(3) J g^−1^) (Figure 2, curve (b)). The physical mixture (PM) of TMA and RSV instead showed a new endothermic peak at ca. 175 °C, due to the melting of a new crystalline phase (curve (c)). The same endothermic peak was present in the KN (curve (d) (and MW, curve not shown) products, preceded by a small exothermic effect at 120 °C, confirming the TMA–RSV interaction and formation of a new thermally-induced solid phase. Comparison of FTIR spectra of the starting components with those of the binary systems showed that several bands shifted to significantly higher frequencies in the treated products, supporting the interpretation based on the thermal data.

With the host TMB (heptakis(2,3,6-tri-O-methyl)-β-CD (TMB)), which displayed in DSC a sharp melting endotherm at *T*_peak,m_ = 158.2(7) °C with Δ*H*_m_ = 38(2) J g^−1^, the TMB–RSV combinations PM and MW yielded products with virtually featureless DSC traces, from which it was deduced that they were amorphous. Given that both the TMB and RSV samples employed were crystalline, with well-defined melting behaviour, it was interesting to note that even physical mixing appeared to yield a significantly amorphous product. (Powder X-ray diffraction of the PM sample confirmed its essentially amorphous nature, though a few prominent peaks due to RSV, of low intensity, were still evident above the general ‘halo’). It was therefore inferred that solid-state interaction had occurred to some extent on physical mixing. For the MW product (amorphous from the PXRD trace), no RSV was evident in the PXRD pattern and solid-state interaction between TMB and RSV was further confirmed from the FTIR spectrum, which showed several peaks displaced to slightly higher wavenumbers.

For the DMB (heptakis(2,6-di-*O*-methyl)-β-CD)–RSV binary combinations, an endotherm at *T*_peak_ = 207.4(5) °C for the preparation PM reflected definite solid-state interaction, but the KN and MW products were effectively amorphous, based on the lack of distinct thermal events. An attempt to recrystallize the PM from MeOH/H_2_O (1:1 v/v) yielded a sample which displayed a distinct endo–exothermic effect in the DSC trace, attributed to inclusion complex formation. The FTIR spectrum of the KN product lacked two characteristic peaks of RSV, suggesting its inclusion in the cavity of DMB. It is noted that the DSC trace from ground single crystals of the phase later identified as the inclusion complex DMB·RSV·4H_2_O instead showed different features from those reported above for DMB–RSV combinations, the most prominent endotherm appearing at ca. 233 °C. We infer that the nature of the inclusion complex formed depends on the preparative method employed.

### Thermal characterization of crystalline CD·RSV inclusion complexes obtained by co-precipitation

The co-precipitation method using small amounts of ethanol to aid dissolution of the RSV produced high-quality single crystals of each of the three inclusion complexes. The host–guest stoichiometries of the inclusion complexes between RSV and the three methylated CDs were all found to be 1:1 from ^1^H NMR spectra of solutions of single crystals of the respective complexes (Supporting Information File 1).

**Figure 3:**
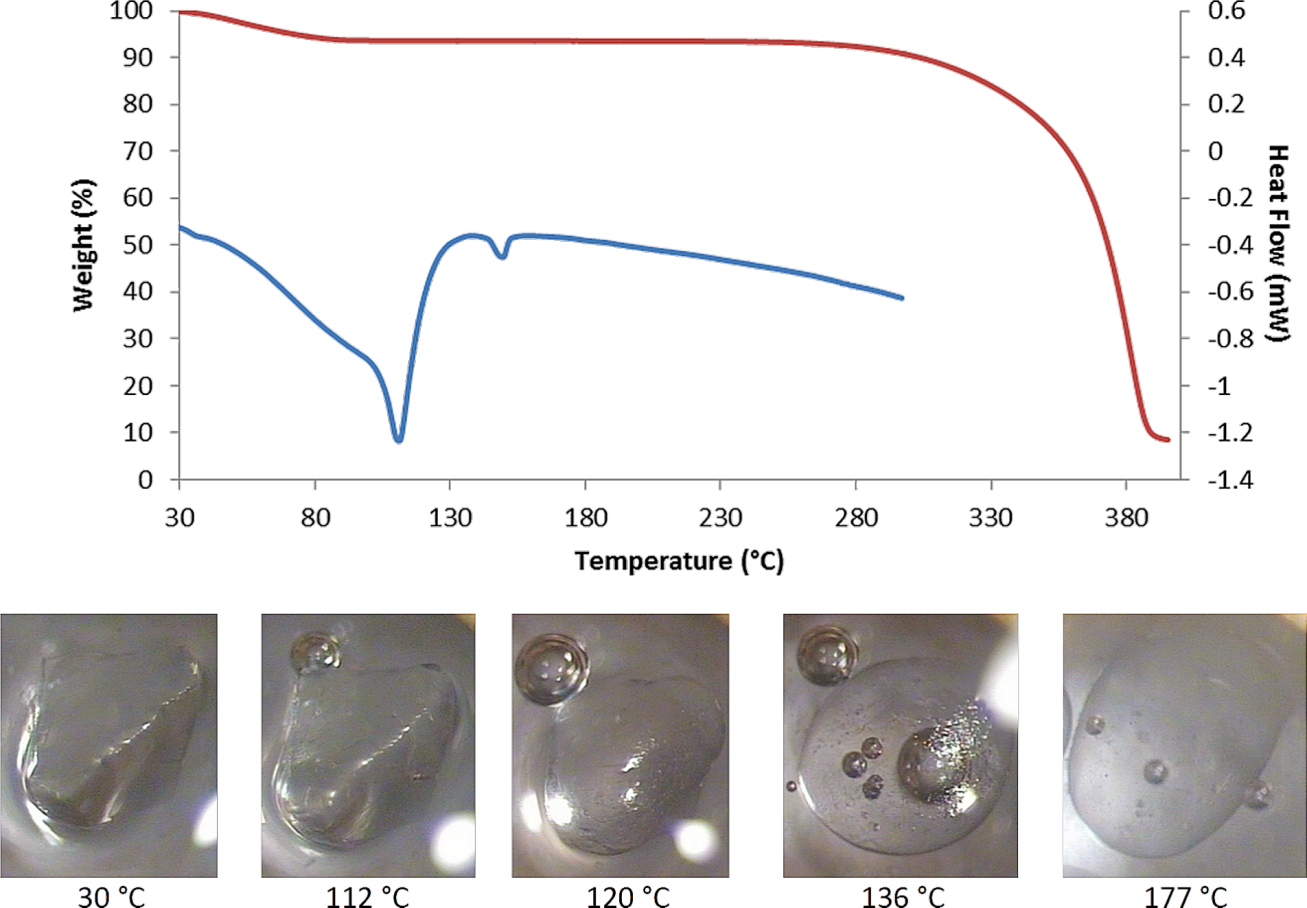
TG (red) and DSC (blue) traces for the hydrated TMA·RSV complex (top), and hot stage micrographs showing the crystals at various temperatures (bottom).

TGA and DSC techniques were used primarily to estimate the water content and/or possible guest loss upon heating and to identify complex melting and other phase changes respectively, with hot stage microscopic (HSM) observations facilitating the interpretation of thermal events. Representative data are shown for the TMA·RSV complex (Figure 3), where a TG mass loss of 7.5 ± 1.3% (*n* = 3) over the temperature range 30–100 °C yielded an estimated 6.6 ± 1.2 water molecules per 1:1 complex unit.

Water loss is evident in the HSM micrograph recorded at 112 °C with the crystal immersed in silicone oil and the DSC trace shows a corresponding broad endotherm accompanying the dehydration. However, a sharp endotherm subsequently developed, peaking at ca. 110 °C, interpreted as commencement of complex fusion which overlaps the dehydration process. This coincides with the melting observed in HSM at 120 °C. A phase change of the anhydrous complex is evident in the HSM at 136 °C, where microcrystallites appear within the melt, the small endotherm at ca. 145 °C being attributed to subsequent melting of the new phase. In HSM, the sample is completely molten at 177 °C. Finally, the TG trace indicates complex decomposition onset at ca. 280 °C.

A summary of the results for the TMB·RSV and DMB·RSV complexes follows (for their TG, DSC and HSM figures, see Supporting Information File 1). The TG trace of the hydrated complex TMB·RSV yielded an initial mass loss of 5.3 ± 0.1% (*n* = 2), equivalent to 5.2 water molecules per 1:1 complex unit. The endotherm observed over the range of 30–120 °C appears sharper than expected for solvent loss alone, suggesting simultaneous melting of the complex. The HSM photographs confirm that dehydration is accompanied by complex fusion, the latter spanning a wide temperature range, with the sample fully molten at 120 °C. Complex decomposition commences at ca. 280 °C. In contrast to TMB·RSV, the thermal behaviour of DMB·RSV is distinctly more complicated (Supporting Information, File 1). The TG trace shows an initial mass loss of 4.4 ± 0.2 % (*n* = 3) over the range 30–110 °C, yielding 4.0 ± 0.2 water molecules per 1:1 complex unit. This loss is reflected in a broad endotherm recorded in the DSC over the same temperature range and is evident in the HSM images from fracturing of the crystal at 130 °C. Between 150 and 200 °C there is negligible mass loss and the anhydrous complex appears to undergo more than one phase transition. A second mass loss appears in the TG trace corresponding to partial guest loss and the DSC shows a sharp but small melting endotherm at ca. 233 °C, the remaining sample decomposing soon after, at ca. 320 °C.

### X-ray analysis

Table 1 lists the crystal data, as well as data-collection and refinement parameters for the new hydrated inclusion complexes TMA·RSV, TMB·RSV and DMB·RSV. The remarkably low *R*_1_*-*factors (range 0.04–0.07) and the relatively small residual electron densities are exceptional for CD structures of this complexity, given also the presence of guest disorder in two cases. An account of the key features of the inclusion of the RSV molecule within the respective host cavities as well as descriptions of the crystal packing arrangements follows.

**Table 1:** Crystal data, data collection parameters and refinement details.

Abbreviated formulae	TMA·RSV·6.25H_2_O	TMB·RSV·5.6H_2_O	DMB·RSV·4.0H_2_O

Complex Formula	C_54_H_96_O_30_·C_14_H_12_O_3_ ·6.25H_2_O	C_63_H_112_O_35_·C_14_H_12_O_3_ ·5.6H_2_O	C_56_H_98_O_35_·C_14_H_12_O_3_ ·4H_2_O
Formula wt. (g mol^−1^)	1566.14	1758.73	1631.71
Crystal system	Monoclinic	Monoclinic	Orthorhombic
Space group	*P*2_1_	*P*2_1_	*P*2_1_2_1_2_1_
*a* (Å)	18.690(9)	10.2142(5)	10.6132(5)
*b* (Å)	21.244(8)	15.2465(8)	15.1612(7)
*c* (Å)	20.528(10)	29.2092(16)	51.066(2)
α (°)	90.0	90.0	90.0
β (°)	94.604(16)	97.1760(10)	90.0
γ (°)	90.0	90.0	90.0
*V* (Å^3^)	8125(6)	4513.1(4)	8216.9(7)
*Z*	4	2	4
*D_c_* (Mg m^−3^)	1.269	1.292	1.319
μ (Mo Kα) (mm^−1^)	0.105	0.106	0.109
F (000)	3316	1886	3496
Data collection temp. (K)	173(2)	173(2)	173(2)
Crystal size (mm)	0.37 × 0.58 × 0.60	0.24 × 0.32 × 0.59	0.19 × 0.31 × 0.37
Range scanned θ (°)	1.7 - 26.4	1.9 - 27.1	1.8 - 26.0
Index ranges ±*h,* ±*k,* ±*l*	−23:15; −26:26; −25:25	−13:13; −19:19; −37:37	−13:6; −18:18; −28:62
Reflections (total)	72298	60403	39193
Independent reflections	17061	10306	8924
Reflections with I > 2σ(I)	13939	9131	7413
No. of parameters	1853	1102	1063
*R*_int_	0.040	0.037	0.047
Goodness-of-fit, *S*	1.027	0.972	1.022
*R*_1_ [*I* > 2σ(*I*)]	0.0677	0.0399	0.0386
Reflections omitted	32	17	9
*wR* on *F*^2^	0.1955	0.1068	0.0887
Weighting scheme *a*, *b* in w = 1/[σ^2^(F_o_^2^) + (aP)^2^ + (bP)]	0.0993, 7.9196	0.0640, 1.4136	0.0439, 1.5448
(Δ/σ)_mean_	< 0.001	< 0.001	< 0.001
Δρ excursions (e Å^−3^)	−0.48 and 0.76	−0.31 and 0.54	−0.25 and 0.32
CCDC no.	1020492	1020493	1020494

**Figure 4:**
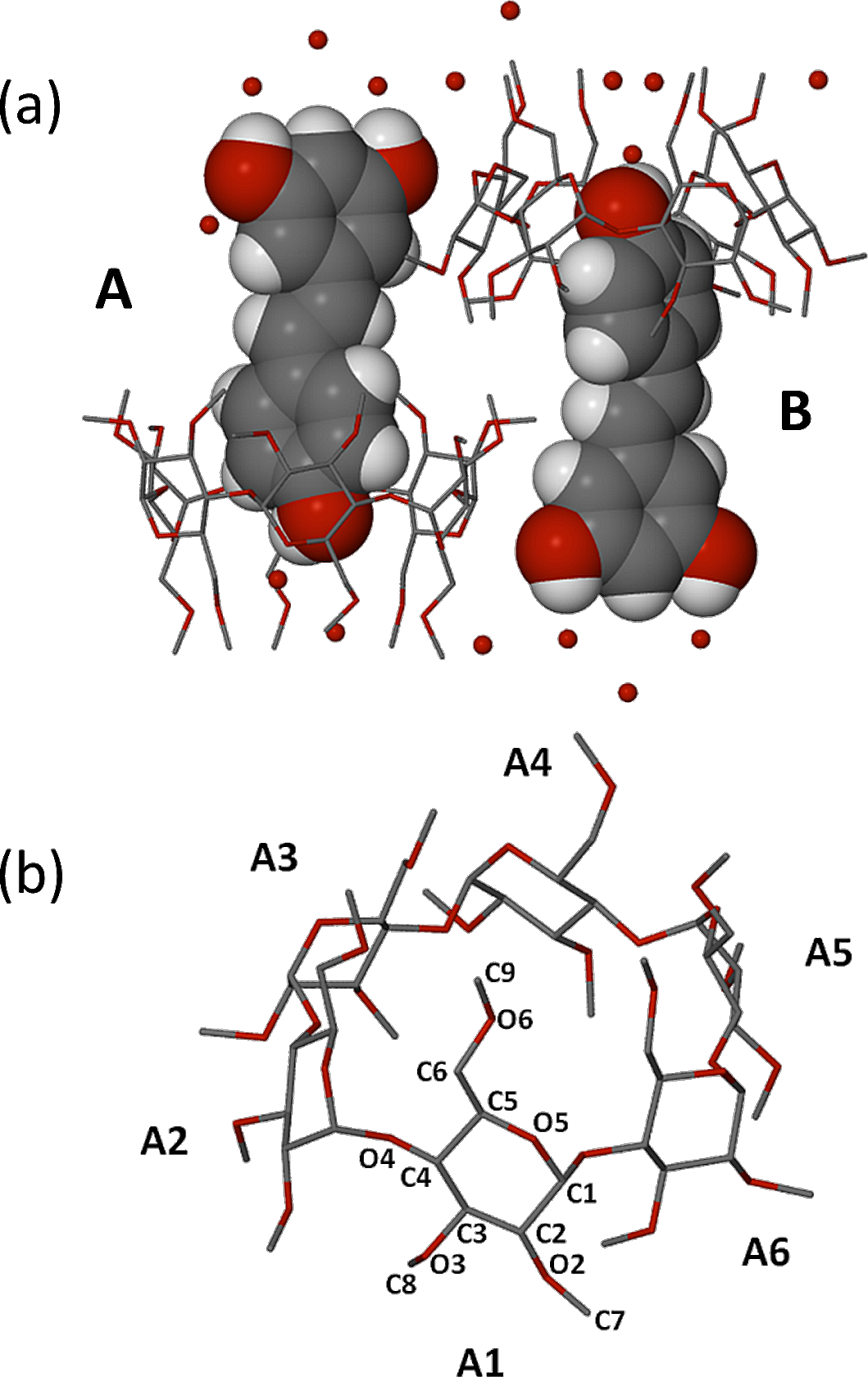
The two symmetry-independent complex units of TMA·RSV·6.25H_2_O (A and B), with only the major component of disorder shown for RSV in host B (a), and the non-H atom and methylglucose ring nomenclature illustrated for host A as representative (b). For clarity, host H atoms have been omitted.

The asymmetric unit of the complex TMA·RSV·6.25H_2_O, namely two TMA molecules, two RSV molecules and 12.5 water molecules, is shown in Figure 4a. In both 1:1 host–guest complex units the guest phenyl ring bearing one phenolic group (the 4-hydroxyphenyl residue) is fully immersed in the host cavity, being located at the primary side, while the ring bearing two phenolic groups (the 1,3-benzenediol residue) protrudes significantly from the host secondary side, where its phenol groups engage in hydrogen bonding with water molecules. Crystallographic atomic nomenlature for the host is shown in Figure 4b.

**Figure 5:**
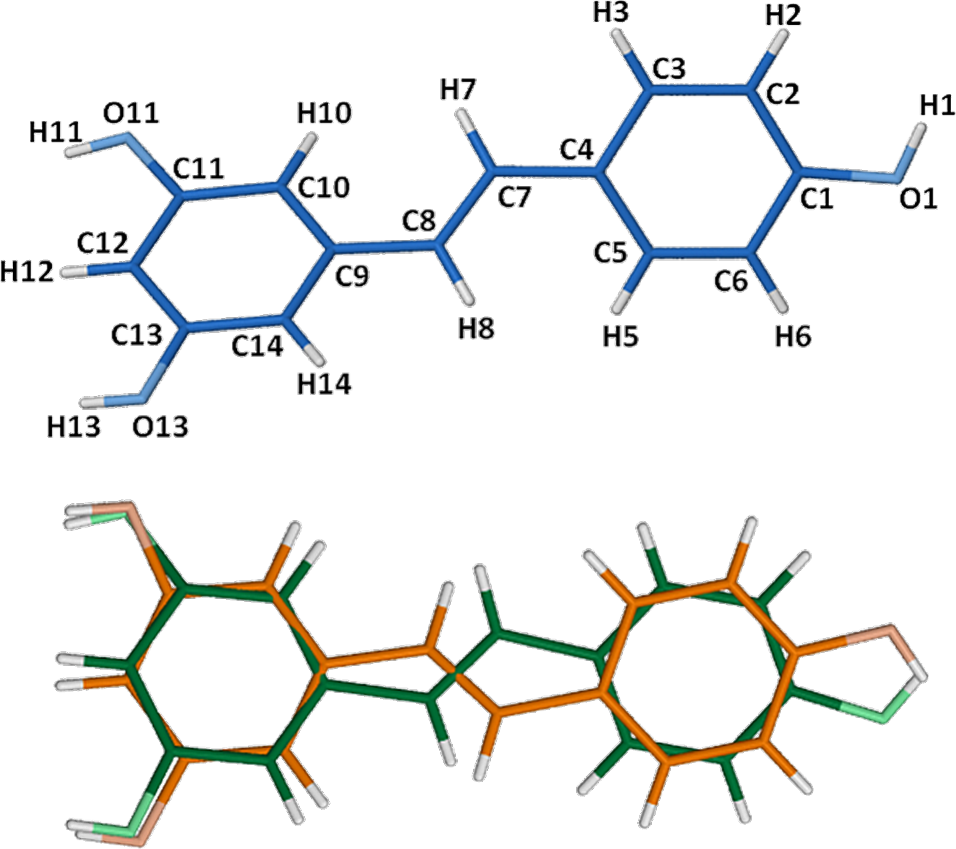
Representative atomic labelling for the ordered RSV molecule A (blue) present in host A and the two disorder components B (orange, s.o.f. = 0.56) and C (green, s.o.f. = 0.44) of the RSV molecule included in host molecule B.

The full description of the guest molecules is provided in Figure 5, where the ordered structure of guest molecule A is contrasted with the twofold-disordered model (components B, C) for the second guest molecule. Several of the host B atoms were disordered over two positions and were modelled accordingly. These included, on the primary side, two C6–O6–C9 chains, a methoxy group and an O5 atom, and on the secondary side, three methoxy groups. Full geometrical analyses that included nine metrical parameters describing the host molecule conformations was performed (Supporting Information, File 1). This revealed that host molecules A and B adopt the expected elliptical shape [12], the longer axis of each macrocycle being approximately parallel to the planes of the respective included 4-hydroxyphenyl rings.

In addition, the crystallographically independent TMA host molecules adopt somewhat different conformations given the fact that their contents differ, owing to the disorder described in Figure 5. In particular, the average extent of ‘tilt’ of each glucose ring relative to the mean O4-plane is small for host molecule A [range 3.24(3)–6.44(5)°], indicating a relatively open primary side, whereas for host B, the average tilt angle is significantly larger [range 5.72(4)–10.31(9)°], reflecting a more ‘closed’ primary side.

Regarding the mode of guest inclusion, the angle between the mean plane of the RSV molecule and the mean O4-plane of the host molecule A is ca. 85.6°, with that between the RSV major disorder component B and the mean O4-plane of host molecule B being virtually the same (ca. 86.8°). While the RSV molecule in its own crystal structure ([10], refcode DALGON) is planar, it is notable that the RSV molecules in the TMA complex deviate significantly from planarity and to different extents; in the case of the ordered RSV guest molecule A, the interplanar angle between the two phenyl residues is 51.6(3)°, and for the major disorder component of RSV which is included in host molecule B, the corresponding angle is 23.1(4)°. Thus, the significant host conformational differences coupled with the significant guest conformational differences reflected in the parameters reported above clearly indicate a mutual induced fit when TMA forms an inclusion complex with RSV. This phenomenon of mutual induced fit has recently been cited as a frequent occurrence in biological systems, but a rare one for synthetic host–guest systems [13]. However, its occurrence in CD inclusion complexes is known and was in recent years prominently manifested in CD complexes of rocuronium salts [14].

**Figure 6:**
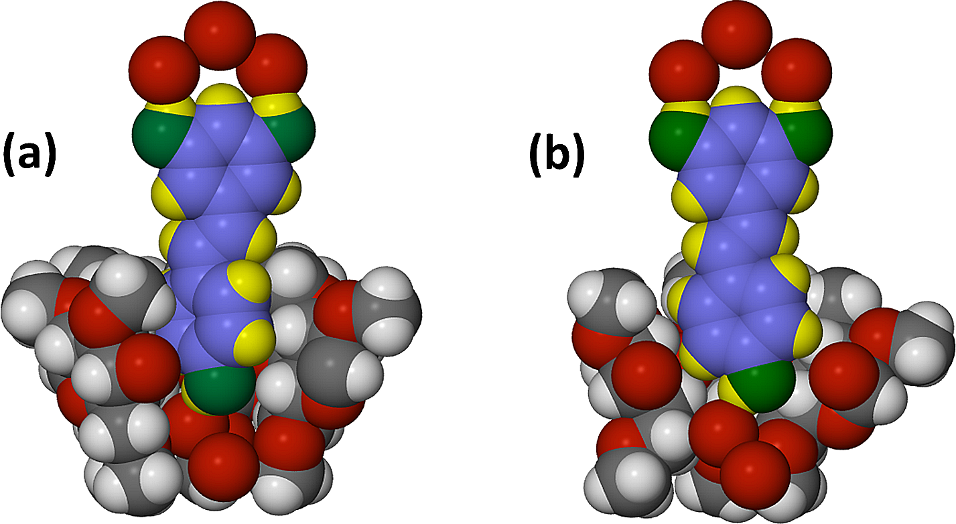
Space-filling representations of the two independent complex units A (a) and B (b) of the complex TMA·RSV·6.25H_2_O with a cutaway view of the host to illustrate the details of guest inclusion. For the RSV molecules, the atoms are colour coded blue (C), green (O) and yellow (H). For clarity, only the major RSV disorder component is shown in (b).

Figure 6 illustrates the three-component supramolecular systems A and B occurring in the crystal. Each consists of a TMA molecule, a RSV molecule and a decorative motif (here referred to as a ‘crown’) of three hydrogen bonded water molecules (H atoms not shown), the terminal water molecules forming hydrogen bonds with the phenolic groups. For the ordered RSV guest in complex A, for example, the four O···O distances are in the range of 2.700(6)–2.863(6) Å. It is noteworthy that the ‘crown’ feature is a robust motif, occurring in all three inclusion complexes described here. Furthermore, this motif is unique to the *trans*-resveratrol inclusion complexes described here: no analogous motifs were found on searching the Cambridge Structural Database [10]. It is also important to note that for the TMA·RSV complex, the major stabilising host–guest interaction is that between the phenolic group of the 4-hydroxyphenyl ring and the primary rim of the host TMA molecule, which is mediated by a bridging water molecule. In ordered complex unit A, for example, the linkage is RSV(4-OH)···O(water)···O6(primary methoxy), with respective O···O distances of 2.731(6) and 2.829(7) Å.

A complex network of hydrogen bonds stabilises the crystal structure; these include host–guest O–H···O and C–H···O hydrogen bonds, host–host C–H···O hydrogen bonds, guest–water and water–water O–H···O hydrogen bonds.

**Figure 7:**
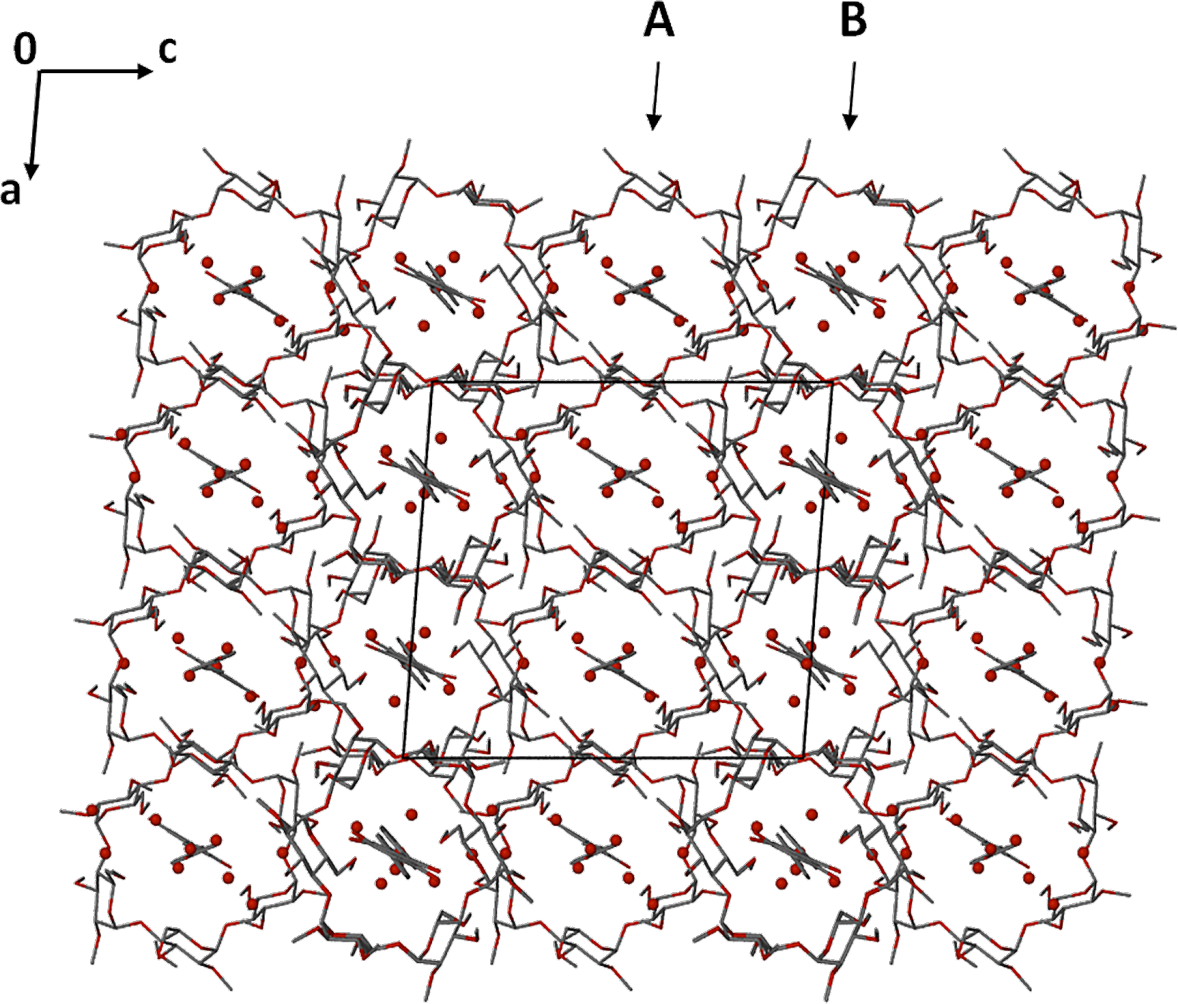
Crystal packing for the complex TMA·RSV·6.25H_2_O projected down [010].

Crystal packing is shown in Figure 7. The complex units pack in a head-to-tail manner in columns parallel to the crystal *b*-axis. Columns of complex units A propagate as rows parallel to the *a*-axis, alternating with analogous columns of B complex units.

**Figure 8:**
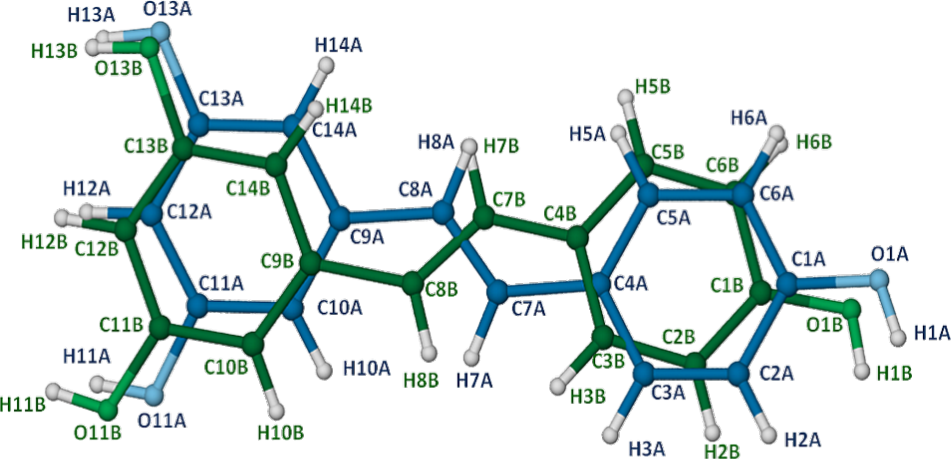
The components of the disorder model for RSV in its inclusion complex with TMB (s.o.f. = 0.73 for the major component A (blue) and 0.27 for the minor component B (green)).

Structural analysis of the inclusion complex between permethylated β-CD (TMB) and RSV, with formula TMB·RSV·5.6H_2_O, revealed twofold disorder of the RSV molecule. The symmetry of the disorder model (Figure 8) is, however, clearly different from that in the TMA complex (Figure 5) but the close proximity of the chemically equivalent phenolic groups of the A and B components in principle enables them to engage in similar hydrogen bonding schemes.

The conformational flexibility of the RSV molecule is again evident in this complex, the interplanar angles between the phenyl rings being 17.7(1)° for the major component and 23.9(3)° for the minor component, thus extending the range of guest conformational flexibility encountered in the TMA complex.

**Figure 9:**
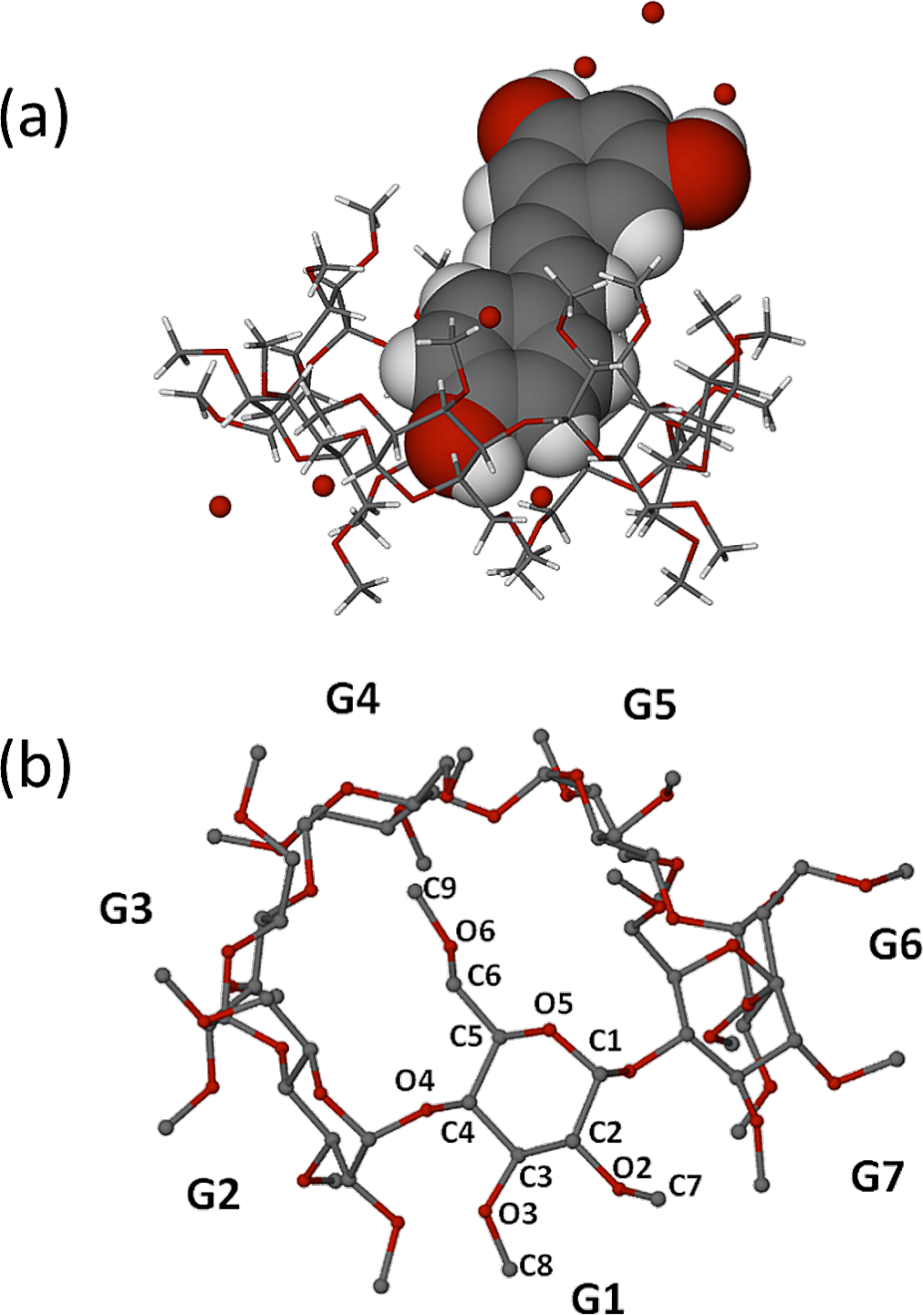
The asymmetric unit in the crystal of TMB·RSV·5.6H_2_O (a), and the non-H atom and methylglucose ring nomenclature illustrated for the host TMB (b). Only the major RSV disorder component is shown in (a) for clarity.

The crystal asymmetric unit contains the equivalent of one RSV molecule, one TMB molecule and 5.6 water molecules (Figure 9). The molecule of RSV is included within the TMB cavity with the 4-hydroxyphenyl group located at the host primary side, being anchored directly via a hydrogen bond [RSV(4-OH)···O611] to a partial oxygen atom (s.o.f. = 0.65) of a primary methoxy group. This differs from the situation in the TMA complex, where the host–guest link is mediated by a bridging water molecule.

The major disorder component of the guest engages in a geometrically more favourable hydrogen bonding interaction, such that the O···O distance in O1A–H1A···O611 is 2.73(1) Å, whereas for the minor guest component, the corresponding O···O distance in O1B–H1B···O611 is 2.95(1) Å. The situation is slightly more complicated since each of the phenolic groups (–O1A–H1A and –O1B–H1B) engages in bifurcated H-bonding, the second acceptor being a disordered water oxygen atom O7W, located at distances 2.60(1) Å and 2.80(1) Å from O1A and O1B respectively.

**Figure 10:**
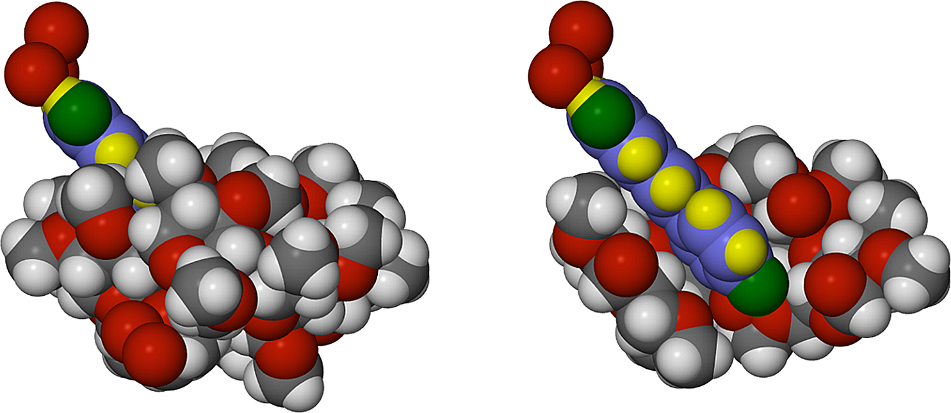
Space-filling model of the inclusion complex TMB·RSV·5.6H_2_O showing the inclusion of the RSV molecule in the host TMB (left) and a cutaway view (right) emphasising the shallow inclination of the guest molecule in the cavity. Only the major guest disorder component is illustrated for clarity. Water hydrogen atoms are also omitted.

Another important feature of the inclusion geometry relates to the guest inclination in the host cavity: here the mean plane of the RSV molecule is inclined at ca. 45° to the mean O4-plane of the TMB molecule (Figure 10), effectively resting on the surface of one side of the host molecule, in strong contrast to the situation in the TMA complex where the equivalent angle is ~86° (Figure 4a). As is usually observed, the primary methoxy groups of the host TMB are generally directed towards the centre of the macrocycle, and effectively close the primary side, presenting a bowl-shaped surface to the RSV molecule. Instead, the secondary side of the host molecule is open and a portion of the 1,3-benzenediol residue protrudes from that side, where the two phenolic groups are again linked by a ‘crown’ of three hydrogen bonded water molecules, analogous to that observed in the TMA complex.

**Figure 11:**
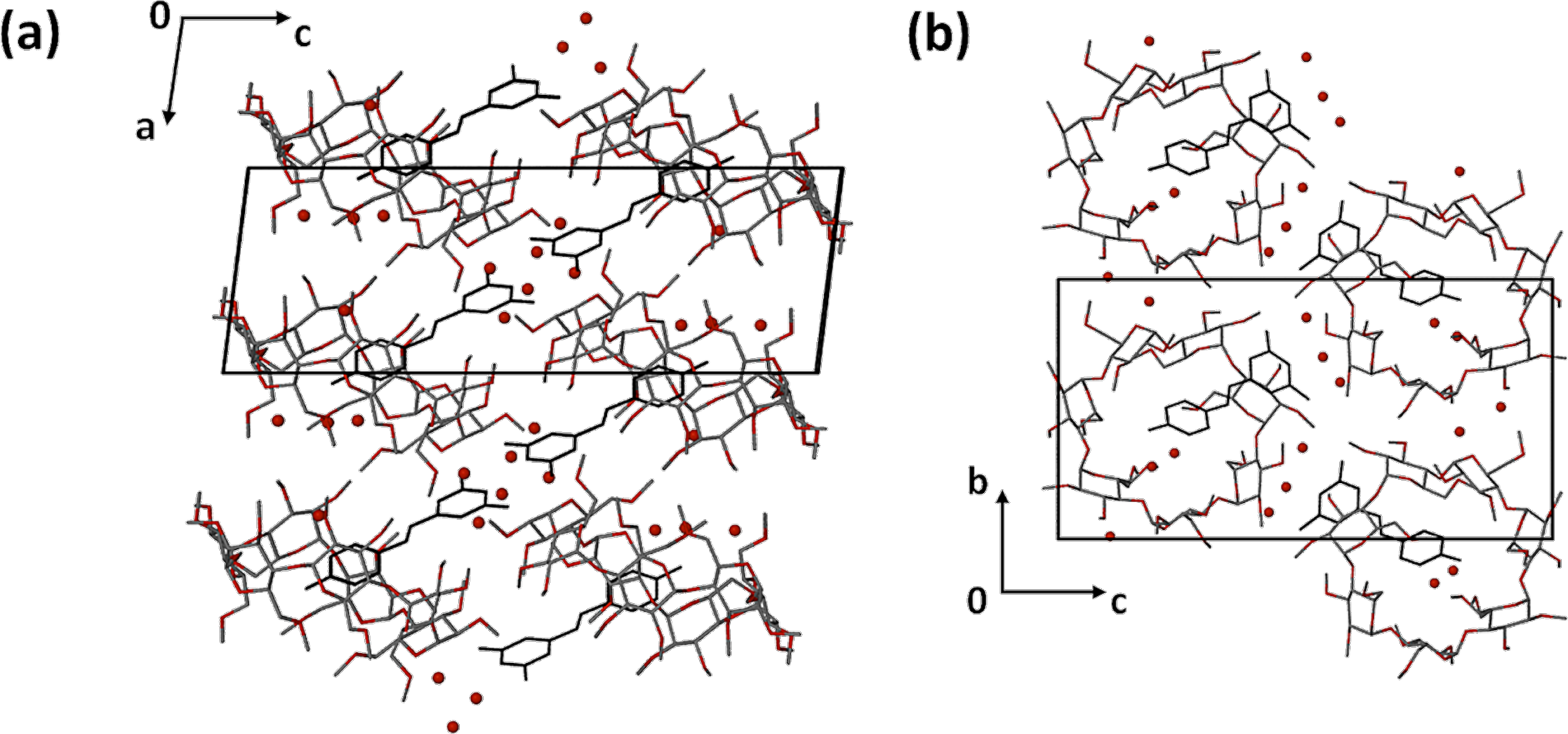
Packing arrangement in the crystal of TMB·RSV·5.6H_2_O viewed down [010] (a) and [100] (b). Hydrogen atoms have been omitted for clarity; water oxygen atoms in red.

The higher quality of the diffraction data for the TMB·RSV complex enabled location of the hydrogen atoms of the water molecules in difference Fourier syntheses. Both disorder components of the RSV molecule engage in equivalent hydrogen bonds with the host molecule. Stabilisation of the crystal structure of TMB·RSV·5.6H_2_O is effected by a complex network of attractive interactions, including host–guest hydrogen bonds (both O–H···O and C–H···O), several host–host C–H···O interactions and numerous O–H···O hydrogen bonds that involve ordered and disordered water molecules (the 5.6 H_2_O molecules in the asymmetric unit being disordered over nine sites). The complex units stack in columns parallel to the *a*-axis in a head-to-tail fashion (Figure 11a), adjacent columns being related by the two-fold screw axis along 1/2, y, 1/2. The view down the columns (Figure 11b) reveals a channel-like arrangement of the host molecules in this direction. Among the various isostructural classes of CD inclusion complexes [15], the one to which this complex belongs has relatively few members.

**Figure 12:**
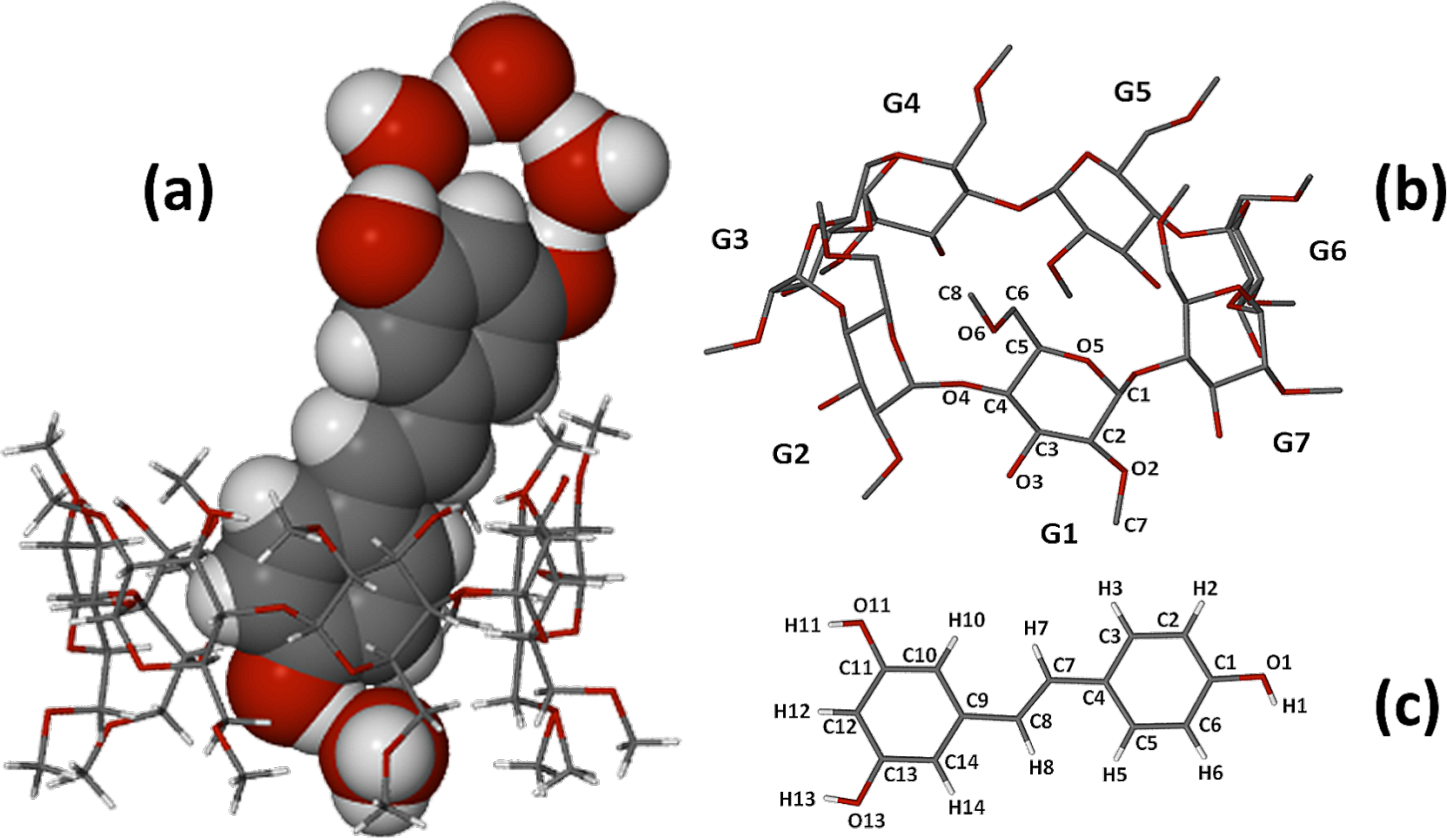
Structure of the host–guest complex DMB·RSV·4.0H_2_O (a), ring and atomic nomenclature for the host molecule DMB (b), and structure and atomic numbering of the included RSV molecule (c).

The third complex whose X-ray structure is described here has the formula DMB·RSV·4.0H_2_O, the host molecule DMB being 2,6-dimethylated β-CD and consequently having properties that are intermediate between those of the native β-CD and fully methylated β-CD [16]. The formula unit corresponds to the crystal asymmetric unit, shown in Figure 12a. Despite the inclusion of the guest molecule, the DMB molecule retains its ‘round shape’ owing to the formation of the well-known ‘belt’ of intramolecular O2(*n*)···O3(*n*−1) hydrogen bonds that link contiguous glucose residues [17–18]. In this complex, the average O···O distance in the belt is 2.83 Å and the O–H···O angles span the range 165–173°.

**Figure 13:**
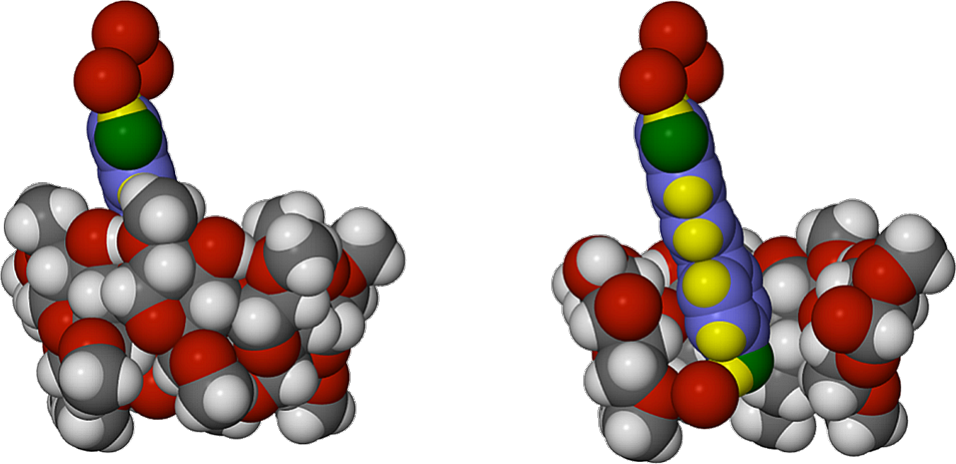
Space-filling model of the inclusion complex DMB·RSV·4.0H_2_O showing the encapsulation of part of the RSV molecule by the host DMB (left) and a cutaway view highlighting the location and orientation of the guest molecule in the host cavity (right).

As in the previous two complexes, the RSV molecule is again included with the 4-hydroxyphenyl ring located deep within the cavity with the phenolic group at the primary side, while the 1,3-benzenediol residue protrudes from the secondary rim of the DMB molecule and the two phenolic groups are again decorated by a ‘crown’ of three hydrogen bonded water molecules. In this complex, the included RSV molecule shows the highest degree of planarity, the phenyl ring planes intersecting at only 13.6(2)°. The topology of guest inclusion is shown in Figure 13. The angle between the mean O4-plane of the DMB molecule and the mean plane through the RSV molecule is ca. 73°, intermediate between the corresponding values in the TMA and TMB complexes.

**Figure 14:**
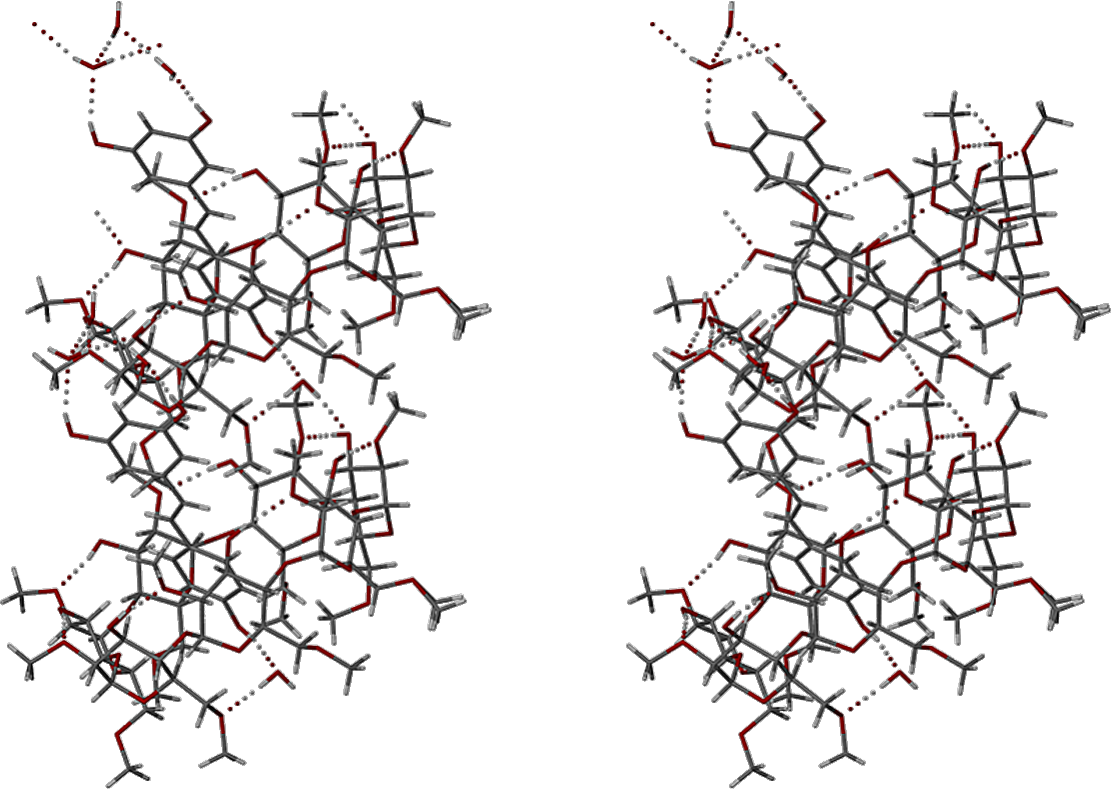
Stereoview of two DMB·RSV·4.0H_2_O complex units related by a unit translation along the crystal *a*-axis, illustrating the intramolecular hydrogen bonds which stabilise the host conformation as well as the hydrogen bonding role of the bridging water molecule that links complex units along the crystal *x*-direction.

Closer examination of the binding of the 4-hydroxyphenyl ring to the host molecule reveals that its hydroxy group is linked to a methoxy oxygen atom (O6G7) on the primary rim of the host molecule via a bridging water molecule, the relevant hydrogen bond sequence being RSV(O1–H1)···O2W–H2WA···O6G7, with respective O···O distances 2.718(4) Å and 2.778(4) Å. The second hydrogen atom on the water molecule (H2WB) is in turn a donor to the atom O3G3^i^ of a translated (i = *−*1 *+ x, y, z*) DMB molecule, this hydrogen bond having a O2W···O3G3^i^ distance of 2.857(3) Å and being responsible for cohesion between successive complex units along the crystal *x*-direction. Figure 14 illustrates the principal hydrogen bonds associated with the two complex units referred to above.

It is noteworthy that in the above motif, the two host molecules are fairly steeply inclined to the *a*-axis (which is approximately vertical) with the result that two primary methoxy groups of the uppermost molecule are partially included within the cavity of the translated molecule. In addition to the hydrogen bonds discussed above, the crystal structure of the DMB complex is stabilised by a series of host–host C–H··· O hydrogen bonds as well as numerous water–water O–H···O hydrogen bonds.

**Figure 15:**
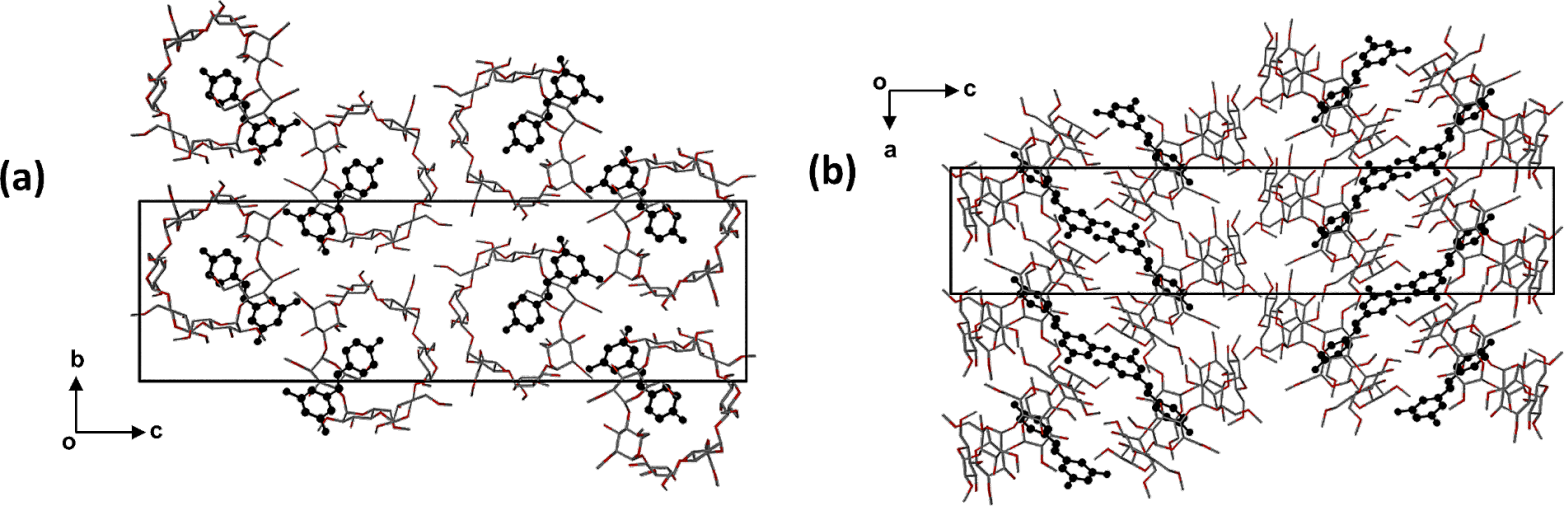
Projections of the crystal structure of the complex DMB·RSV·4H_2_O along [100] (a) and [010] (b). Hydrogen atoms are omitted for clarity.

The crystal packing is shown in Figure 15. Complex units stack in columns parallel to the *a*-axis in a head-to-tail fashion with (as noted above) a small extent of host self-inclusion (Figure 15a). Figure 15b illustrates the modified herringbone packing arrangement as viewed down the *b*-axis.

Regarding the phase purity of the three new inclusion complexes described above, we confirmed that their simulated powder X-ray diffraction patterns are in good agreement with those calculated from the single crystal X-ray data. This is an important verification that the single crystals selected are truly representative of the respective bulk materials (Supporting Information File 1).

### Phase-solubility analysis

According to Higuchi and Connors [11], phase-solubility diagrams can be classified as being of types A and B. A-type behaviour corresponds to an increase in the solubility of the drug as the concentration of the CD is increased, as a result of soluble complex formation. A-type curves can further be distinguished depending on whether the solubility increases linearly (A_L_) as the CD concentration increases, or with a positive (A_P_-type) or negative (A_N_-type) deviation due to a change in the physical properties of the solution. B-type curves indicate the formation of an insoluble complex, where B_S_ suggests the formation of a complex with limited solubility, while B_I_ denotes the formation of an insoluble complex.

**Figure 16:**
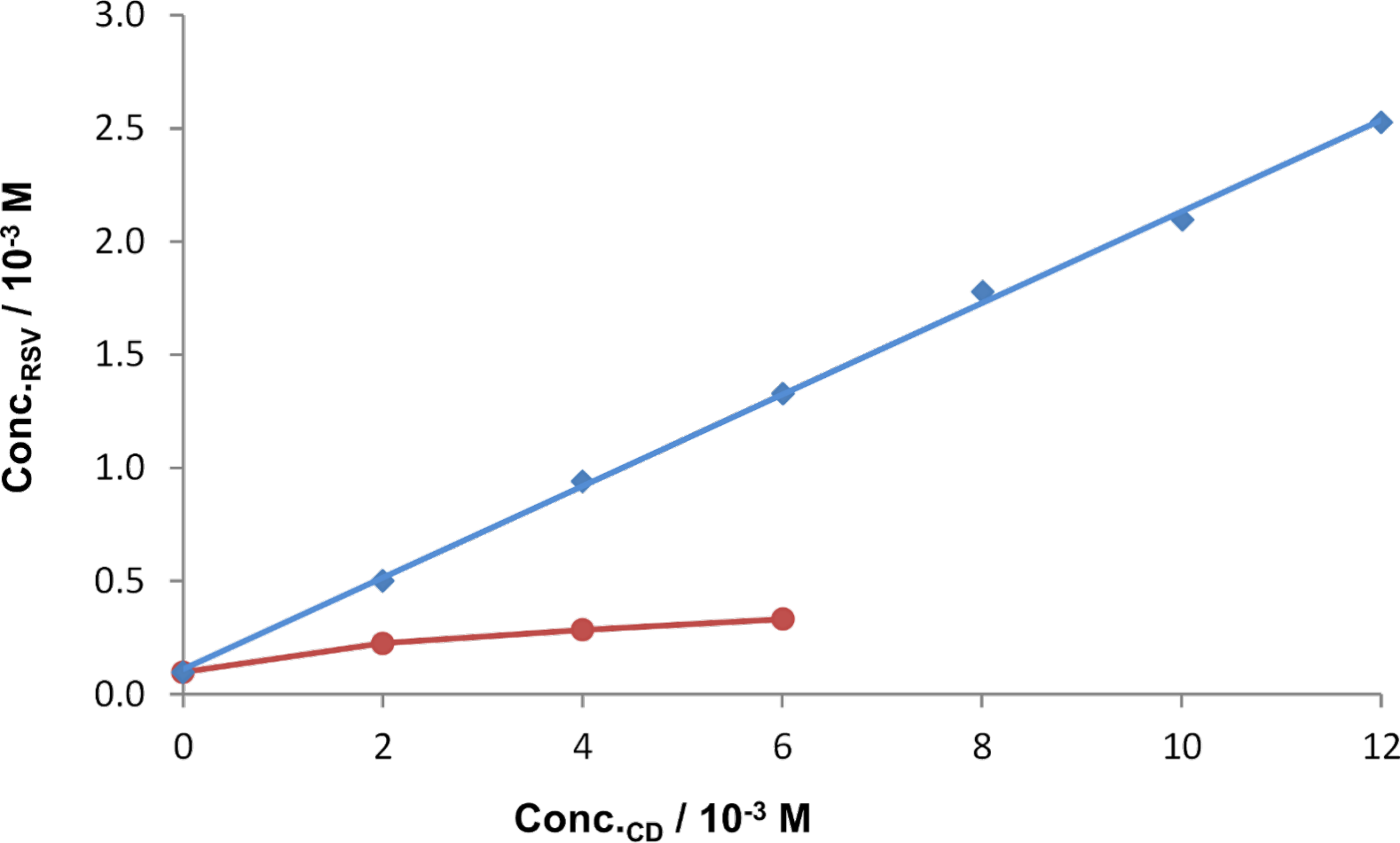
Solubility of RSV as a function of [β-CD] (blue) and [γ-CD] (red) at 25 °C.

Figure 16 shows the phase-solubility results for RSV with the native CDs β- and γ-CD. The phase-solubility profile resulting from the use of β-CD is of type A_L_ and this host produces a guest solubility enhancement of 26-fold over the concentration range indicated. The results for the experiments with γ-CD were limited to a maximum CD concentration of 6 mM by inefficient filtration through the filter membrane that was employed. The precipitation of complex or aggregated CD particulates was physically observed during sample preparation. Over this range the solubility plot appears to increase to a plateau, indicating an A_N_ solubility profile, the negative deviation possibly being due to changes in the solubility of the complex and/or aggregation of the CD molecules. The solubility enhancement for RSV with γ-CD was only 3.4-fold.

**Figure 17:**
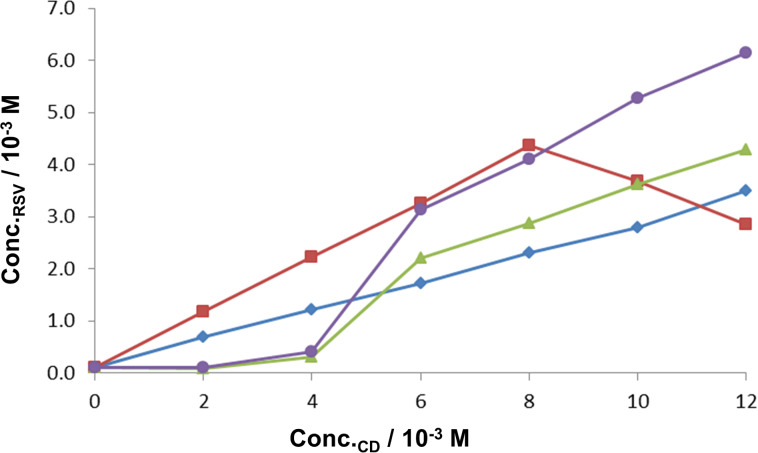
Solubility of RSV as a function of the concentrations of TMB (light blue), DMB (red), HP-β-CD (green) and RMB (dark blue) at 25 °C.

The solubility enhancements for RSV in the presence of the derivatised CDs are significant (Figure 17). With TMB, A_L_-type behaviour was observed with a solubility enhancement of 36 times that of the intrinsic solubility of the guest. Each of the remaining derivatised CDs shows two different solubility profiles over the common concentration range. Hydroxypropyl-β-CD (HP-β-CD) and randomly methylated β-CD (RMB) show relatively small initial solubility enhancements of RSV solubility (up to ca. 4 mM CD concentrations), with significant solubility increases thereafter (A_L_-type). The changes in slope may indicate an increase in the complex order with respect to RSV. The solubility enhancement for RSV at the highest CD concentration employed is 44-fold in the presence of HP-β-CD and 63-fold in the presence of RMB.

The results with DMB follow the opposite trend, with the solubility of the guest increasing linearly over the CD concentration range 0–8 mM, while above that concentration, the apparent solubility of RSV decreases. This is attributed to the formation of an insoluble complex, which removes RSV from the solution. The maximum solubility enhancement, occurring at a CD concentration of 8 mM is 45 times that of the intrinsic solubility of the guest.

Values of the association constants for complex formation (*K*_C_) were estimated using the relationship (1) and the slopes of the recorded phase-solubility diagrams, assuming 1:1 host–guest complex formation [11].

[1]
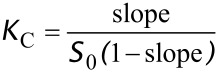


Table 2 shows the approximate stability constants for complexation between each of the CDs investigated and RSV. Only the initial slopes were used to calculate *K*_C_ (up to 6 mM for γ-CD, 8 mM for DMB and 4 mM for HP-β-CD and RMB).

**Table 2:** The apparent stability constants (*K*_C_) for complexation between various CDs and RSV.

Cyclodextrin	*K*_C_ [M*^−^*^1^]

β-CD	2600
γ-CD	410
TMB	3900
DMB	11600
HP-β-CD	580
RMB	890

The values obtained indicate relatively weak interactions between RSV and γ-CD, initially weak interaction with HP-β-CD and RMB, fairly strong binding with β-CD and TMB, and the formation of a very stable complex between RSV and DMB up to the CD concentration of 8 mM.

Lu et al. [8] found a linear relationship between the concentration of RSV and the concentrations of both β-CD and HP-β-CD, reporting the derived *K*_C_ values as 1815 M^−1^ for β-CD·RSV and 6778 M^−1^ for HP-β-CD·RSV. The conditions under which these experiments were carried out were slightly different from ours, however: an excess of RSV was added to 5 mL of CD solution; the solutions were shaken for 24 h and the suspensions were filtered with cellulose acetate. The results we obtained for the phase-solubility behaviour using β-CD are comparable to those obtained by Lu et al. [8], but the results obtained with HP-β-CD are quite different, probably due to the different preparation methods used. However, a comparison of the data for the derivatised CDs TMB and DMB show a similarly strong interaction to that obtained by Lu et al. with HP-β-CD. The general trend, indicating that the derivatised CDs interact more strongly with RSV, is confirmed in the present study as well.

Regarding the reliability of the data based on this methodology when dealing with RSV solutions, we noted that the choice of filtration membrane in these studies can greatly affect the outcome of the experiment. We found that nylon filters remove RSV from the aqueous solution completely, allowing only some of the molecules which are protected by the CD to pass through the filter membrane. Of all the membranes tested the PTFE filters were found to give the most consistent results, although cellulose acetate was not tested in the present study.

## Conclusion

A variety of methods (physical mixing, kneading, microwave irradiation) of effecting interaction between RSV and three CD hosts (TMA, DMB and TMB) was tested and a combination of thermal analysis and FTIR spectroscopy subsequently yielded evidence for the formation of interaction products, many of them amorphous in nature. For more definitive characterization of interaction products, crystalline inclusion complexes with the formulae TMA·RSV·6.25H_2_O, TMB·RSV·5.6H_2_O and DMB·RSV·4.0H_2_O were subsequently isolated using the co-precipitation method and fully characterized by thermal and single crystal X-ray diffraction methods. For the complexes containing the fully methylated hosts TMA and TMB, thermal analysis revealed dehydration overlapping with fusion of the anhydrous complex, followed by final decomposition, whereas the DMB complex displayed more intricate thermal events, namely dehydration followed by phase transitions and partial guest loss that preceded final decomposition.

The X-ray studies reported here reveal, for the first time, the unique features of the mode of inclusion of the RSV molecule within CDs. The TMA complex contains two symmetry independent TMA·RSV complex units: in one of these, the guest is ordered while in the second the guest is disordered over two positions. This disorder was successfully modelled, as was the slightly modified twofold disorder of the RSV molecule in the TMB·RSV complex, and in all cases, the disorder never results in spatial interchange of the 4-hydroxyphenyl and 1,3-benzenediol units; instead, it brings the 4-hydroxyphenyl residues of the two disordered components into close proximity, and likewise the respective 1,3-benzenediol residues. DMB·RSV is the only complex in which there is no guest disorder.

For all three hosts, complex formation involves insertion of the less sterically bulky 4-hydroxyphenyl ring of RSV deep within the CD cavity where it is located at the host primary side. In the TMA and DMB complexes, the phenolic group is linked to the host by hydrogen bonding of the type RSV(4-OH)···O(water)···O6(primary methoxy), whereas in the TMB complex, there is a direct host–guest linkage via a RSV(4-OH)···O6(primary methoxy) hydrogen bond. A common feature, however, is the significant extent of protrusion of the 1,3-benzenediol moiety from the secondary side of each of the three hosts, with the two phenolic groups being linked by a series of four hydrogen bonds RSV(1-OH)···O(water)···O(water)···O(water)···[HO(-3)-RSV]. This persistent supramolecular motif has not been observed previously in the solid state and since it may also exist in aqueous solution, it is worth consideration in molecular modelling studies that address CD-RSV interaction in that medium. Equally significant in the context of molecular modelling is our observation in the solid-state of the potentially more probable bridging role of water in mediating host–guest binding, this feature occurring in two of the three complex crystal structures investigated. Finally, as far as further new insights from the X-ray studies are concerned, we conclude that CD-RSV inclusion in the more flexible hosts TMA and TMB involves a mutual induced fit. The evidence for this is the flexibility displayed by the RSV molecule, reflected in the wide range observed for the interplanar angle between the phenyl rings [17.7(1)–51.6(3)°] in the respective complex crystal structures, coupled with significant host distortions to accommodate the RSV molecule. In contrast, with the host DMB, whose round structure is maintained by intramolecular hydrogen bonds, the resulting unrestricted cavity volume enables the RSV molecule to be accommodated with very little adaptation, the interplanar angle between the phenyl rings being only 13.6(2)°.

Phase-solubility studies have been useful in confirming the generally higher solubility enhancements that derivatised CDs confer on RSV. Derived values of the association constants for 1:1 CD–RSV complexation in aqueous solution spanned the range of 410 M^−1^ for inclusion in γ-CD to 11 600 M^−1^ for inclusion in DMB.

## Experimental

### Materials

The *trans*-resveratrol sample used for the preparation of binary mixtures was a generous gift from Denk Feinchemie GmbH (München, Germany). For co-precipitation experiments, the RSV used was supplied by Sigma-Aldrich (South Africa). Cyclodextrins were purchased from Wacker Chemie Italia Srl (Milan, Italy) and Cyclolab (Budapest, Hungary). All other materials and solvents used were of analytical reagent grade.

### Preparation of the binary systems

Each physical mixture (PM) (1:1 mol/mol) was prepared by gentle co-grinding of the powder components in a mortar with a pestle and passing the resultant material through a 250 μm sieve.

Kneaded products (KN) were prepared by wetting each PM in a mortar with ethanol/water 4:1 (v/v) and grinding thoroughly with a pestle, after which the product was dried to constant weight at 70 °C in an oven. The entire procedure was repeated in triplicate. The samples were then sieved through a 250 μm sieve.

Co-evaporated products (CP) were prepared by dissolving each PM in the minimum amount of ethanol/water 4:1 (v/v) to obtain a clear solution. The solvent was removed using a rotavapor under reduced pressure at 80 °C, and the residue was gently ground in a mortar with a pestle, and passed through a 250 μm sieve.

Microwave irradiation products (MP) were prepared by dissolving each PM in the minimum amount of ethanol/water 4:1 (v/v) to obtain a clear solution in a glass container, followed by microwave irradiation at 425 W (Pabish CM-Aquatronic) for a time sufficient to remove the solvent. The dried residue was gently ground in a mortar with a pestle, and passed through a 250 μm sieve.

### Differential scanning calorimetry (DSC) and thermogravimetric analysis (TGA)

For the binary systems investigated, temperature and enthalpy values were measured with a Mettler STAR^e^ system (Mettler Toledo, Novate Milanese, MI, Italy) equipped with a DSC821^e^ Module and an Intracooler device for sub-ambient temperature analysis (Julabo FT 900) on 2–4 mg (Mettler M3 Microbalance) samples in sealed aluminium pans with pierced lid [heating rate β = 10 K min^−1^, nitrogen atmosphere (flux 50 mL min^−1^), 30–350 °C temperature range)]. The instrument was previously calibrated with indium as standard reference. Measurements were carried out at least in triplicate. For co-precipitated, crystalline products, traces were recorded on a DSC-Q200 differential scanning calorimeter with samples in closed aluminium pans heated at 10 K min^−1^ and dry nitrogen purge gas flowing at 50 mL min^−1^. TG traces for these products were recorded on samples in alumina crucibles using a TA-Q500 instrument under similar conditions as for the DSC measurements.

### Simultaneous thermogravimetric analysis (TGA/DSC)

Mass losses were recorded with a Mettler STAR^e^ system (Mettler Toledo, Novate Milanese, MI, Italy) TGA with simultaneous DSC (TGA/DSC1) on 4–6 mg samples in alumina crucibles with lid [β = 10 K min^−1^, nitrogen air atmosphere (flux 50 mL min^−1^), 30–350 °C temperature range]. The instrument was previously calibrated with indium as standard reference and measurements were carried out at least in triplicate.

### Fourier transform infrared (FTIR) spectroscopy

Mid-IR (650–4000 cm^−1^) spectra were recorded on powder samples using a Spectrum One Perkin-Elmer FTIR spectrophotometer (resolution 4 cm^−1^) (Perkin Elmer, Wellesley, MA, USA) equipped with a MIRacle^TM^ ATR device (Pike Technologies, Madison, WI, USA).

### Crystal preparation

*trans*-Resveratrol (20 mg) was dissolved in 0.5 mL of ethanol and was added to an equimolar amount of CD dissolved in water, according to Table 3 below. Turbid solutions were clarified by adding ethanol dropwise. Each solution was then filtered into a new vial, closed with a punctured polytop lid and was allowed to evaporate slowly on the benchtop or in an oven. The vial was sealed after crystals appeared.

**Table 3:** Masses of CDs employed, volumes of water added and temperatures at which complex crystals formed.

Cyclodextrin	Mass (mg)	Volume (mL)	Temperature (°C)

Hexakis(2,3,6-tri-*O*-methyl)-α-CD (TMA)	107.3	1	20
Heptakis(2,3,6-tri-*O*-methyl)-β-CD (TMB)	125.2	3	60
Heptakis(2,6-di-*O*-methyl)-β-CD (DMB)	116.6	3	60

### X-ray diffraction analysis

All intensity data were collected on a Bruker KAPPA APEX II DUO diffractometer. In each case a single crystal was surface-dried, coated in paratone N oil (Exxon Chemical Co., TX, USA) and mounted on a cryoloop in a constant stream of nitrogen vapour (Oxford Cryostream, UK). Crystal systems and space groups for the CD complexes were deduced from the Laue symmetries and systematic absences, respectively. The structures were solved by direct methods (program SHELXD [19]) and refined by full-matrix least-squares (program SHELXH-97 [19]). In general, location of the host molecules from the *E*-map was followed by their refinement using isotropic thermal displacement parameters. This was followed by location of the guest molecules from the resulting difference Fourier synthesis. Successive difference maps revealed the water molecules, which were modelled with appropriate site-occupancy factors (s.o.f.s) to reconcile the model with the thermogravimetric analytical data as far as possible. Disorder of the host and guest residues, where they occurred, were similarly treated using appropriate s.o.f.s. In the final cycles of refinement, anisotropic thermal displacement parameters were introduced for most or all of the non-H atoms. A large proportion of the H atoms were located in difference Fourier syntheses and were generally included in idealised positions in a riding model with *U*_iso_ values in the range 1.2–1.5 times those of their parent atoms. Further details of the refinements for the individual complexes appear in the Supporting Information File 1. CCDC 1020492–1020494 contain the supplementary crystallographic data for this paper. These data can be obtained free of charge at http://www.ccdc.cam.ac.uk/products/csd/request/ [or from the Cambridge Crystallographic Data Centre (CCDC), 12 Union Road, Cambridge CB2 1EZ, UK; fax: +44 (0)1223-336033; email: deposit@ccdc.cam.ac.uk].

### Phase-solubility analysis

Phase-solubility studies were peformed according to the method described by Higuchi and Connors [11]. Six CDs [β-CD, γ-CD, TMB, DMB, HP-β-CD and RMB] were dissolved in water to yield solutions whose concentrations spanned the range 2.0–12.0 × 10^−3^ M. An excess of RSV (1.5–2.5 mg) was added to 2 mL of each CD solution and the solutions were allowed to stir at 25 ± 0.5 °C for 48 h. The solubility of RSV in the absence of CD (S_o_) was determined by preparing solutions containing an excess of RSV in water, and stirring at 25 ± 0.5 °C for 48 h. Samples were subsequently filtered through 0.45 μm PTFE syringe filters and diluted appropriately. The concentration of RSV was determined using UV–vis spectrophotometry at a wavelength of 316 nm. The UV spectra were recorded on a GCB Cintra 20 UV–vis spectrometer over a wavelength range of 200–500 nm at a scanning rate of 200 nm min^−1^. The extinction coefficient was determined for this wavelength by preparing a calibration curve of RSV in water. All measurements were recorded in triplicate. Each phase-solubility curve was prepared by plotting the concentration of RSV against the concentrations of the CD employed in the experiment.

## Supporting Information

Additional experimental data include thermal (HSM, DSC, TGA) and FTIR data for CD–RSV combinations, thermal data (HSM, DSC, TGA) for the crystalline complexes, ^1^H NMR peak integrations for complex stoichiometry determinations, details of X-ray structural refinements, geometrical data for CD host conformations, and comparative experimental and calculated PXRD patterns for the complexes.

File 1 Additional experimental data.
