# Supplementary material for: Inclusion of trans-resveratrol in methylated cyclodextrins: synthesis and solid-state structures
Source: Beilstein J Org Chem. 2014 Dec 29;10:3136–51. doi: 10.3762/bjoc.10.331 (PMC4311586; doi:10.3762/bjoc.10.331)
Supplement: File 1 — Additional experimental data. [file Beilstein_J_Org_Chem-10-3136-s001.pdf]

SUPPORTING INFORMATION FILE 1

Inclusion of *trans*-resveratrol in methylated cyclodextrins: synthesis and solid-state structures

Lee Trollope[1], Dyanne L. Cruickshank[1], Terence Noonan[1], Susan A. Bourne[1], Milena Sorrenti[2], Laura Catenacci[2] and Mino R. Caira*[1]

Address:

[1] Centre for Supramolecular Chemistry Research (CSCR), Department of Chemistry, University of Cape Town, Rondebosch 7701, South Africa

[2] Department of Drug Sciences, University of Pavia, Via Taramelli 12, 27100 Pavia, Italy

CONTENTS:

1. Thermal characterization of *trans*-resveratrol (RSV)

2. FT-IR data for TMA-RSV interaction

3. DSC and FT-IR data for TMB-RSV interaction

4. DSC and FT-IR data for DMB-RSV interaction

5. ^{1}H NMR integration for complex host-guest stoichiometries

6. Thermoanalytical characterization of single crystals of the TMB·RSV complex

7. Thermoanalytical characterization of single crystals of the DMB·RSV complex

8. X-ray crystal structure refinement details.

9. Geometrical data defining the conformations of the host molecules

10. Experimental and simulated PXRD patterns for the inclusion complexes

1. Thermal characterization of *trans*-resveratrol (RSV)

Commercial resveratrol (RSV) is a microcrystalline white powder with a thermal profile typical of an anhydrous drug. In the DSC curve of commercial RSV, only one endothermic effect appeared at $T_{peak,m}$ = 266.3(4) °C ($T_{onset,m}$ = 265.1(3) °C; ΔH_m = 279(2) J g^{-1}) due to the drug melting. The anhydrous nature of commercial RSV was confirmed by TGA analysis that revealed mass loss at 275 °C attributable to sample decomposition.

To further characterize the solid state of RSV, it was analysed by FT-IR spectroscopy and the spectrum obtained presents a broad band at 3184 cm^{-1} due to the O-H stretching vibration, a set of typical bands at 1605, 1581 and 1383 cm^{-1} corresponding to C-C aromatic double bond stretching, C-C olefinic stretching and C-O stretching, respectively. The band at 965 cm^{-1} is attributable to the *trans* olefinic bond.

Commercial RSV was treated by kneading and microwave irradiation, the same techniques used to prepare binary systems with cyclodextrins, and the thermic and enthalpic parameters of the solid phases obtained are reported in Table S1. Compared with the untreated material, the absence of differences in enthalpic and thermal parameters confirmed that the same solid phase of RSV was retained. By means of the decomposition temperatures extrapolated by TGA measurements, it was possible to confirm the physical stability of RSV to the treatments used.

Table S1. Thermal data for commercial RSV compared to RSV treated by kneading (KN) and microwave irradiation (MW). (Standard deviation in parentheses (*n*=3)).

RSV	$T_{onset,m}$ (°C)	$T_{peak,m}$ (°C)	ΔH_m (J g^{-1})	T_{dec} (°C) (by TGA)
commercial	265.1(3)	266.3(4)	279(2)	274.6(4)
KN	265.4(1)	266.2(1)	266(1)	270.2(3)
MW	265.3(2)	266.3(1)	265(3)	270.3(9)

2. FT-IR data for TMA-RSV interaction

Comparison of FT-IR spectra of RSV, TRIMEA and their binary system shows significant shifts of several bands (in particular 1581, 1605, 2813, 2920 cm^{-1}) just evident in the physical mixture but more pronounced at higher wave numbers in the treated products (1590, 1606, 2819, 2929 cm^{-1} respectively) supporting the interaction revealed by thermal data.

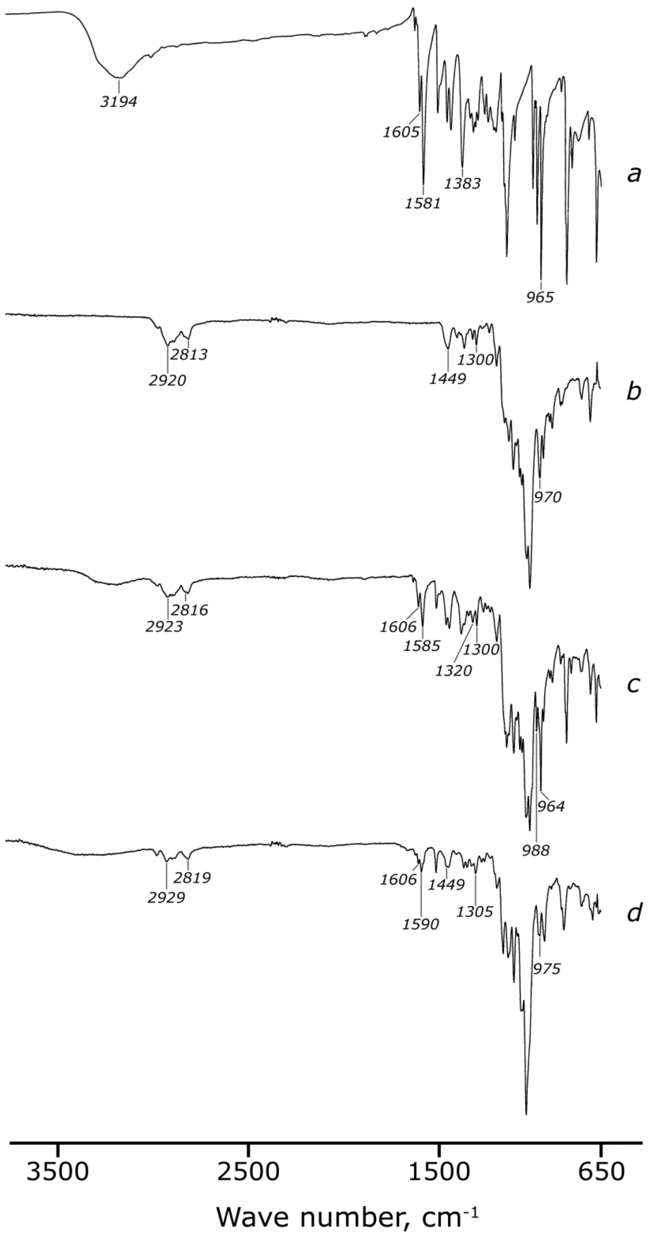

Figure S1. FT-IR spectra of RSV (a), TMA (b), RSV-TMA 1:1 physical mixture, (c) product from kneading RSV and TMA (d).

3. DSC and FT-IR data for TMB-RSV interaction

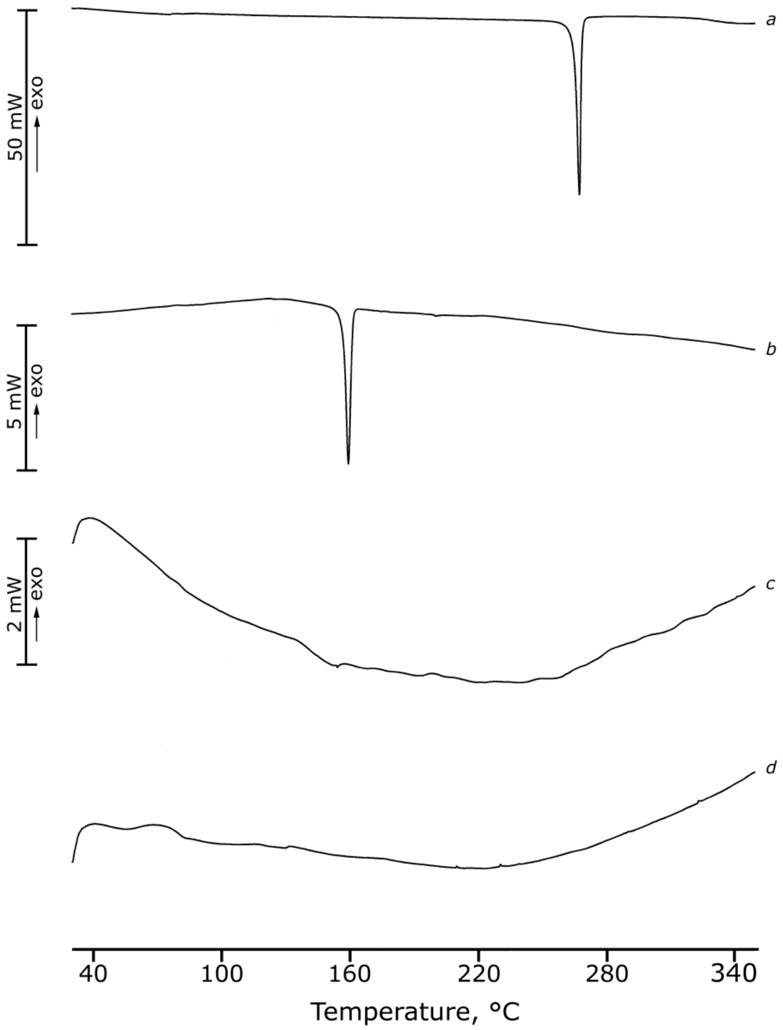

Figure S2. DSC curves for RSV (a), TMB (b), RSV-TMB 1:1 physical mixture (c), product from microwave irradiation treatment (d).

Both RSV and the cyclodextrin TMB are crystalline, the latter showing a melting endotherm at $T_{peak,m}$ = 158.2 ± 0.7 °C (ΔH_m = 38 ± 2 J g^{-1}) in the DSC profile (curve *b*). The simple mixing process induced amorphisation of this binary system owing to an interaction in the solid state between drug and carrier, confirmed also in the product obtained by MW irradiation (curves *c* and *d*). Evidence for a small residual of crystalline material was evident from peaks in the PXRD pattern at 2θ-values of 27.4 and 31.8°.

The thermal data are supported by FT-IR analysis (Figure S3). The shifts of some bands to higher wave numbers in the spectrum of the microwave product confirm the solid state interaction.

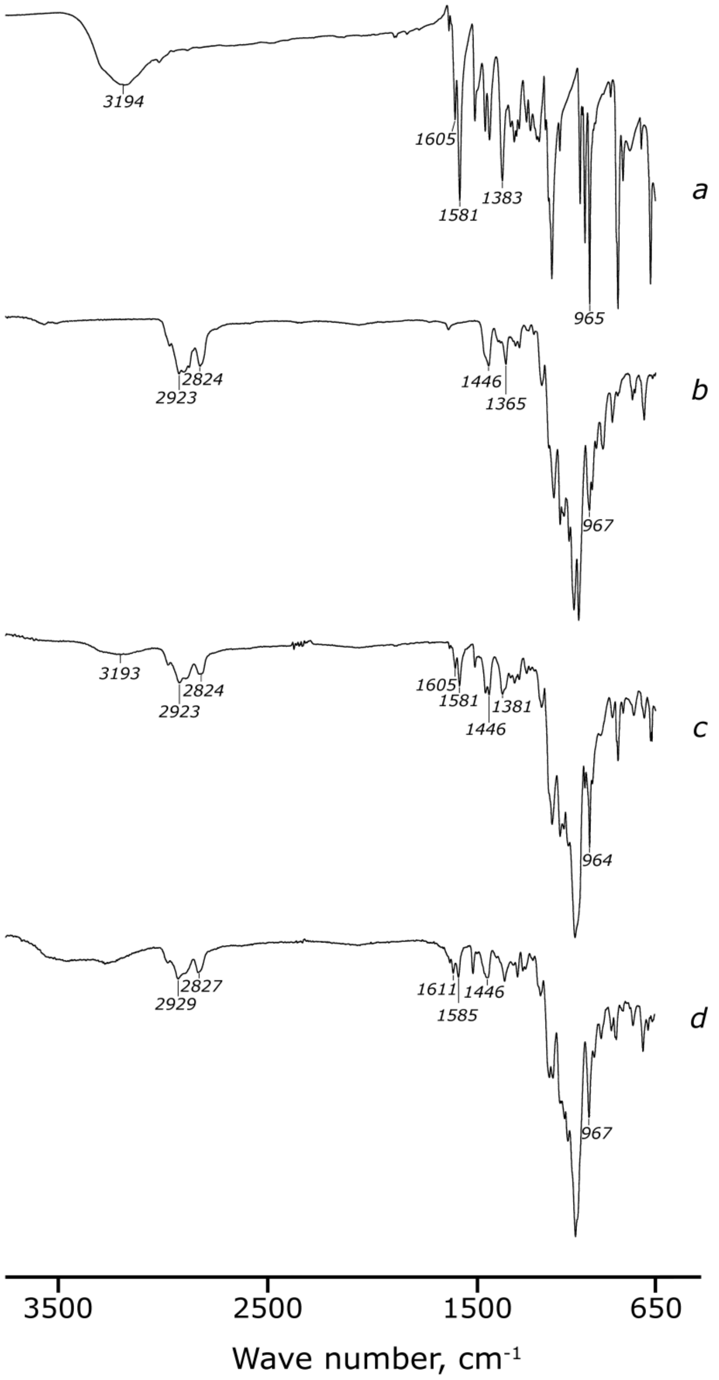

Figure S3. FT-IR spectra of RSV (a), TMB (b), RSV-TMB 1:1 physical mixture (c), product from microwave irradiation treatment (d).

4. DSC and FT-IR data for DMB-RSV interaction

In the DSC profile of the physical mixture (Figure S4, curve *c*) an endothermic effect appears at T_{peak} = 207.4 ± 0,5 °C due to a solid state interaction between the drug and DMB by simple physical mixing; this interaction in the KN (curve *d*) and MW (curve not shown) products is evident as a complete amorphisation of the system. The appearance of an endo-exothermic effect in the DSC curve of the system obtained by crystallization (curve *e*) is attributed to the inclusion of the drug in the CD cavity.

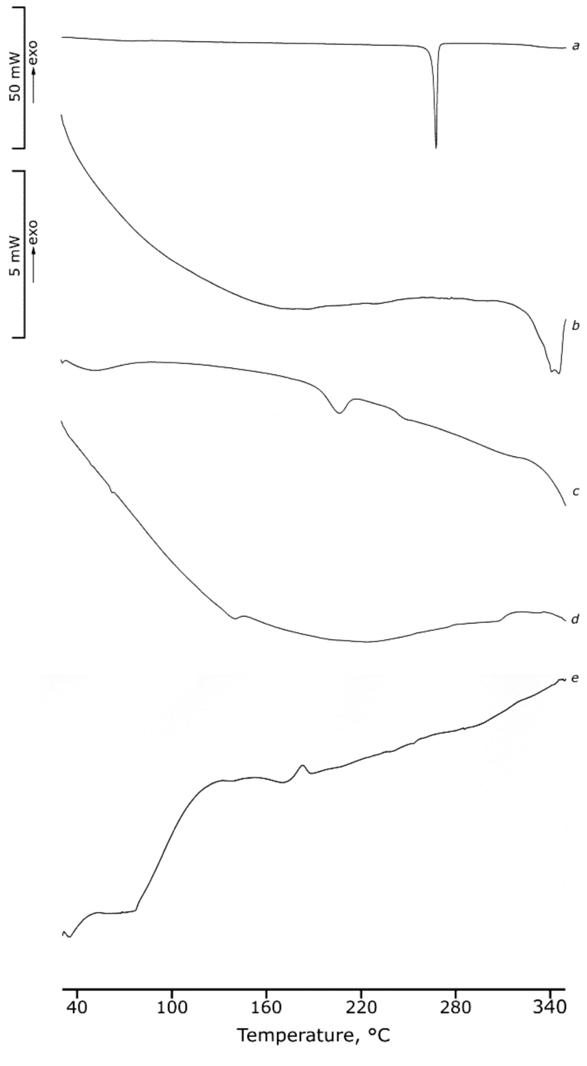

Figure S4. DSC curves for RSV (a), DMB (b), RSV-DMB 1:1 physical mixture (c), product from kneading (KN) (d), the product obtained by crystallization of the 1:1 physical mixture from a MeOH/H_2O (1:1 v/v) solution (e).

The solid-state interaction between the drug and cyclodextrin is confirmed by FT-IR spectra of the treated physical mixtures. As an example, in Figure S5 the spectrum (*d*) of the KN product is reported, from which it is evident that the characteristic bands of RSV at 1605 and 1581 cm^{-1} that are still present in the spectrum (*c*) of the physical mixture no longer appear as a resolved doublet.

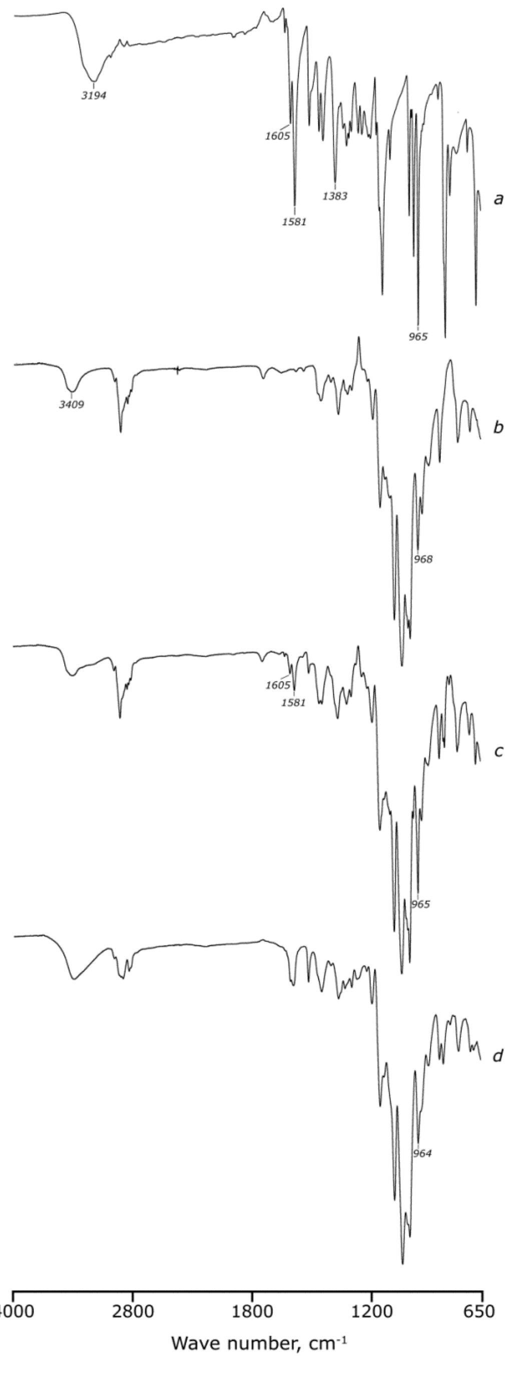

Figure S5. FT-IR spectra of RSV (a), DMB (b), RSV-DMB 1:1 physical mixture (c), product obtained by kneading the physical mixture (d).

5. ^{1}H NMR integration for complex host-guest stoichiometries

Following selection of single crystals of the inclusion complexes, the specimens were thoroughly surface-dried on filter paper, dissolved in acetone-d_6 (TMA·RSV, TMB·RSV) and DMSO-d_6 (DMB·RSV) and their ^{1}H NMR spectra were recorded on a 400MHz Bruker AMX 400 spectrometer. The data were analysed using the Bruker software Topspin, Mestronova or MestRe-C. Proton integration data confirming the 1:1 host-guest stoichiometries are tabulated below. The atomic labelling schemes are appended to the table.

Table S2. Integrals of the NMR signals for the protons of dissolved inclusion complexes employed in the determination of host-guest complex stoichiometries.

TMA·RSV

Proton	δ (ppm)	Multiplicity	J (Hz)	Integration	Experimental/ Theoretical
2 x C-$\underline{H}_B$	7.435	d	^3J = 8.33	1.00	1.00
C-$\underline{H}_C$ + C-$\underline{H}_D$	6.965	d of d	^4J = 16.29	1.94	0.97
2 x C-$\underline{H}_A$	6.859	d	^3J = 8.65		0.97
2 x C-$\underline{H}_E$	6.557	d	^3J = 2.15	1.00 *	1.00
C-$\underline{H}_F$	6.288	t	^4J = 2.17	0.46	0.92
6 x C-$\underline{H}_1$	5.036	d	^3J = 3.45	3.07	1.02
6 x O-C$\underline{H}_3$	3.613	s		9.26	1.02
6 x O-C$\underline{H}_3$	3.355	s		9.23	1.02
6 x C-$\underline{H}_5$	3.069	d of d	^4J = 3.43	3.13	1.04

TMB·RSV

Proton	δ (ppm)	Multiplicity	J (Hz)	Integration	Experimental/ Theoretical
2 x C-$\underline{H}_B$	7.435	d	^3J = 8.73	0.99	0.99
C-$\underline{H}_C$ + C-$\underline{H}_D$	6.965	d of d	^4J = 16.34	1.90	0.95
2 x C-$\underline{H}_A$	6.859	d	^3J = 8.65		0.95
2 x C-$\underline{H}_E$	6.557	d	^3J = 2.15	1.00 *	1.00
C-$\underline{H}_F$	6.288	t	^4J = 2.17	0.46	0.92
7 x C-$\underline{H}_1$	5.148	d	^3J = 3.68	3.61	1.03
7 x O-C$\underline{H}_3$	3.352	s		10.45	1.00
7 x C-$\underline{H}_5$	3.111	d of d	^4J = 3.67	3.69	1.05

DMB·RSV

Proton	δ (ppm)	Multiplicity	J (Hz)	Integration	Experimental/ Theoretical
p-OH	9.515	s		0.96	0.96
2 x m-OH	9.159	s		2.00	1.00
2 x C-$\underline{H}_B$	7.399	d	^3J = 8.62	2.02	1.01
C-$\underline{H}_C$ + C-$\underline{H}_D$	6.873	d of d	^4J = 16.32	1.93	0.97
2 x C-$\underline{H}_A$	6.763	d	^3J = 8.64	2.06	1.03
2 x C-$\underline{H}_E$	6.387	d	^3J = 2.13	2.05	1.02
C-$\underline{H}_F$	6.124	t	^4J = 2.13	1.00 *	1.00
6 X C-$\underline{H}_1$	4.982	d	^3J = 3.68	14.72	1.05
C-O$\underline{H}$	4.958	s			1.05
2 x C-$\underline{H}_6$	3.582	d	^3J = 3.14	14.75	1.05
6 x O-C$\underline{H}_3$	3.519	s		22.47	1.07
6 x O-C$\underline{H}_3$	3.269	s		22.65	1.08
C-$\underline{H}_5$	3.218	d of d	^3J = 3.58	7.61	1.08

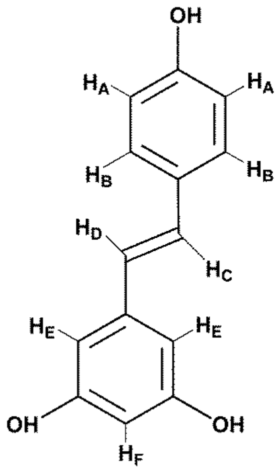	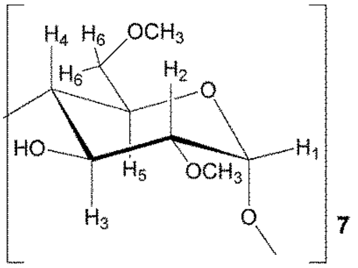	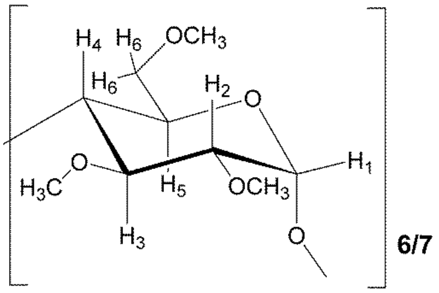
trans-Resveratrol (RSV) labelling	DMB labelling	TMA/TMB labelling

6. Thermoanalytical characterization of single crystals of the TMB·RSV complex

Figures showing the thermogravimetric, differential scanning calorimetric and hot stage microscopic data for the complex TMB·RSV·5.6H_2O are provided below. Interpretation of the data appears in the manuscript.

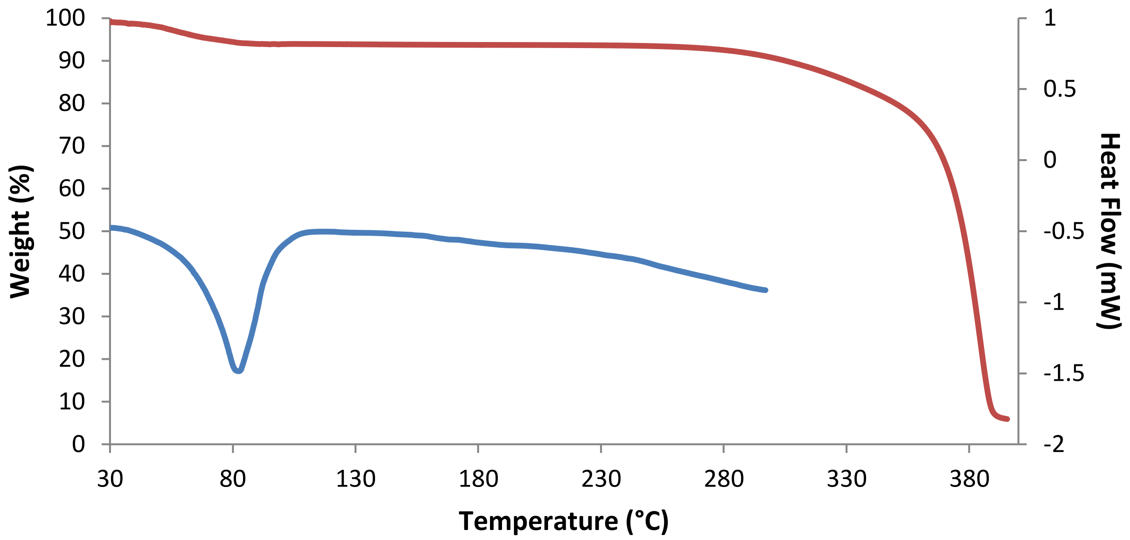

Figure S6. TGA (red) and DSC (blue) traces for the complex TMB·RSV·5.6H_2O.

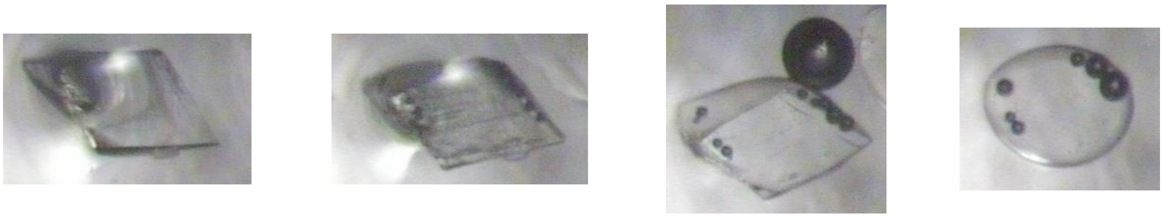

30 °C 90 °C 96 °C 120 °C

Figure S7. Hot stage micrographs for the complex TMB·RSV·5.6H_2O.

7. Thermoanalytical characterization of single crystals of the DMB·RSV complex

Figures showing the thermogravimetric, differential scanning calorimetric and hot stage microscopic data for the complex DMB·RSV·4.0H_2O are provided below. Interpretation of the data appears in the manuscript.

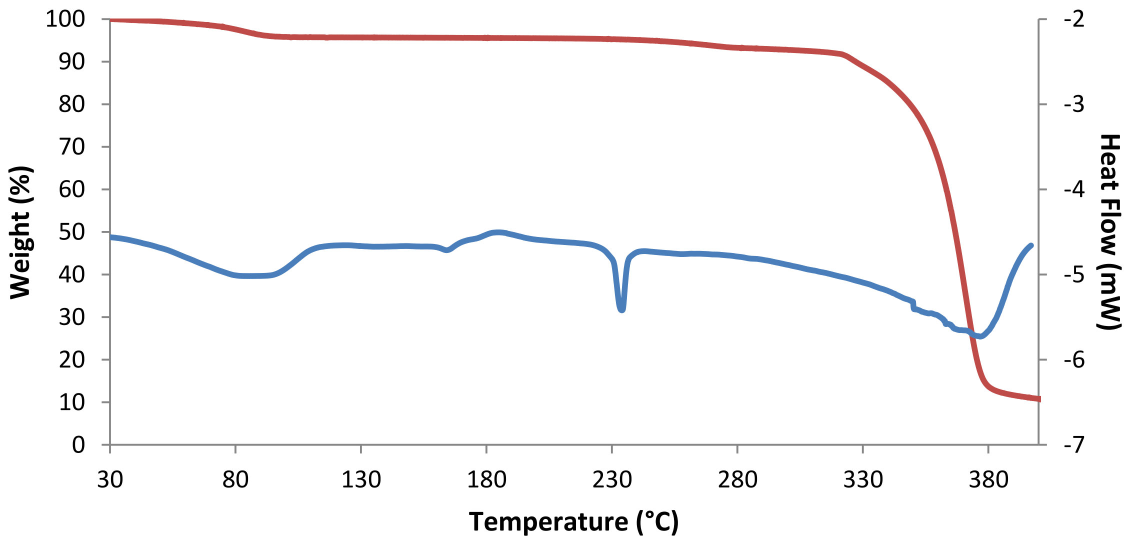

Figure S8. TGA (red) and DSC (blue) traces for the complex DMB·RSV·4.0H_2O.

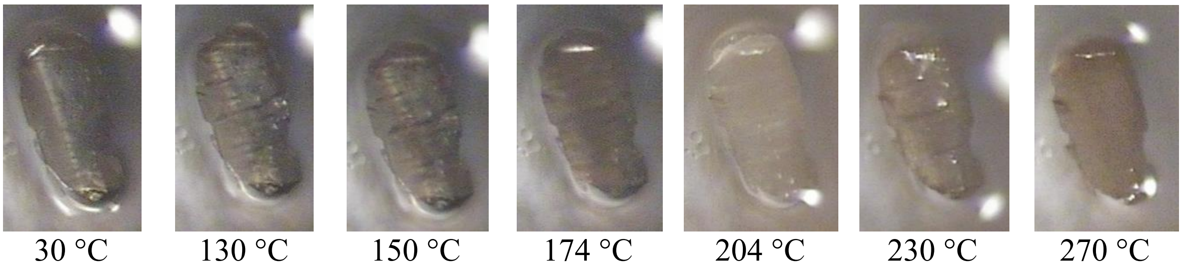

Figure S9. Hot stage micrographs for the complex DMB·RSV·4.0H_2O.

8. X-ray crystal structure refinement details.

Complex TMA·RSV·6.25H_2O

The asymmetric unit was found to contain two TMA molecules (A, B) and two RSV molecules. The glucopyranose units in the respective host molecules were distinguished with labels A and B. Several atoms of the host molecule B were found to be disordered and these were assigned site-occupancy factors (s.o.f.s) of x and 1-x, where x is variable. Table S3 shows the disordered pairs and their s.o.f.s.

Table S3. Parameters for the disordered atoms on the TMA host molecule B.

<table>
<tr><th>Atom</th><th>s.o.f.</th><th>Atom</th><th>s.o.f.</th><th>Atom</th><th>s.o.f.</th></tr>
<tr><td>C721/C722</td><td>0.41/0.59</td><td rowspan="4">C631/C632
O631/O632
C931/C932</td><td rowspan="4">0.49/0.51</td><td rowspan="4">O561/O562
C561/C562
C661/C662
O661/O662
C961/C962</td><td rowspan="4">0.55/0.45</td></tr>
<tr><td>C751/C752</td><td>0.58/0.42</td></tr>
<tr><td>C761/C762</td><td>0.55/0.45</td></tr>
<tr><td>O651/O652
C951/C952</td><td>0.83/0.17</td></tr>
</table>

The remaining significant electron density peaks not associated with either a host or guest molecule were assigned as water molecules. A total of 12.5 water molecules were assigned. This is in good agreement with the TGA results, which revealed a loss of 13.2 ± 2.4 (n = 3) water molecules per asymmetric unit. The latter were assigned over 20 sites, eight of which were fully ocupied (s.o.f.s = 1.0) while 12 sites were treated as being partially occupied. The U_{iso} values of the water molecules refined to values between 0.04 and 0.11 Å^2 with a mean value of 0.06 Å^2. The hydrogen atoms of the water molecules were not located.

The non-hydrogen atoms with unit s.o.f.s were refined anisotropically; all disordered non-hydrogen atoms were refined isotropically. Several bond distances involving the disordered atoms were constrained to appropriate values in the range 1.40(2)-1.50(2) Å.

All of the host tertiary, secondary and methyl hydrogen atoms were placed in idealised positions and refined in a riding model. H atoms were assigned U_{iso} values in the range 1.2-1.5 times those of their parent atoms.

Modelling the *trans*-resveratrol guest

Electron density peaks found within the host molecule A were assigned as the non-hydrogen atoms of guest molecule A. No disorder was evident and the U_{iso} values were found to be stable. The non-hydrogen atoms were subsequently refined anisotropically. The guest molecule included in CD host molecule B was found to be disordered over two positions. After some effort, the disordered components (labelled B and C) were successfully modelled. The phenyl rings of these components were constrained as rigid hexagons using the AFIX 66 instruction in SHELXH-97. The s.o.f.s of the disordered guest components refined to 0.56 for B and 0.44 for C. The aromatic hydrogen atoms were added in idealised positions in a riding model. The guest hydroxyl hydrogen atoms were placed in idealised positions using the commands AFIX 83 and AFIX 147. The former uses the hydrogen bond searching model while the latter identifies the idealised position by the highest electron density using a rotating group model. These hydroxyl H atoms refined to reasonable positions, consistent with the expected hydrogen bonding scheme.

Complex TMB·RSV·5.6H_2O

The primary methoxy fragment on glucopyranose unit G1 was found to be disordered over two positions. The disordered components were refined with partial s.o.f.s of x and 1 – x respectively. The s.o.f. of the major fragment C611-O611-C911 refined to 0.65. The ordered non-hydrogen atoms displayed reasonable U_{iso} values and were allowed to refine anisotropically. Many of the hydrogen atoms appeared in the difference Fourier map; however, all were placed in idealised positions in a riding model. They were assigned U_{iso} values in the range 1.2-1.5 times those of their parent atoms.

Isolated electron density peaks were assigned as water molecules. Atoms O4W and O5W were found to be disordered over two positions. The U_{iso} values for O6W and O7W were abnormally high indicating partial occupancy. An average U_{iso} was applied to these and their s.o.f.s were allowed to refine freely. Once the s.o.f.s had settled, these were fixed and the U_{iso} values were allowed to refine freely. Electron density peaks corresponding to the expected positions of the hydrogen atoms of the water molecules were identified and assigned for atoms O1W, O2W, O3W and

O4W. The positions of the hydrogen atoms were fixed using the DANG and DFIX commands. A total of 5.6 water molecules were assigned over 9 sites (Table S4 lists the atoms and their s.o.f.s). This number of modelled water molecules is in reasonable agreement with the TGA result of 4.0 ± 1.4.

Table S4. The site-occupancy factors assigned to water oxygen atoms.

Atom	s.o.f.	Atom	s.o.f.
O1W	1.00	O5W1	0.50
O2W	1.00	O5W2	0.50
O3W	1.00	O6W	0.31
O4W1	0.73	O7W	0.29
O4W2	0.27		

Modelling the *trans*-resveratrol guest

The guest phenyl ring was apparent in the first difference Fourier synthesis based on the host atoms as trial model. Subsequent refinements revealed the remainder of the RSV molecule. Further refinements revealed guest disorder over two positions, with the disorder appearing as a result of rotation about the C4-C7 and C8-C9 bonds, such that the double bond is "criss-crossed". The s.o.f. of the major component (A) was refined as x while the minor component (B) was refined as 1-x. The final s.o.f. of A refined to 0.73. Only the major component A was allowed to refine anisotropically. The AFIX 66 command was applied to the phenyl rings to constrain them to idealised hexagonal geometry. The aromatic hydrogen atoms were added in idealised positions in a riding model. The hydroxyl hydrogen atoms were placed using a hydrogen bond searching model. H atoms were assigned U_{iso} values in the range 1.2-1.5 times those of their parent atoms.

Complex DMB·RSV·4.0H$_2$O

Many of the host and guest non-H atoms were located in the E-map. Following their refinement with isotropic thermal displacement parameters, successive difference Fourier maps revealed the remaining non-hydrogen atoms. Neither of the guest phenyl rings required restraints and refined freely throughout. The glucopyranose

units were labelled G1-G7 and disordered atoms were labelled by parts. The methyl carbon on the O6 methoxy group of glucopyranose unit G2 displayed abnormally high thermal motion and was found to be disordered over two positions. The major component refined with a s.o.f. of 0.55 and the minor component with s.o.f. 0.45. The C-O bond lengths in the disordered methyl groups O6G2-C821 and O6G2-O822 were restrained to a length of 1.40(2) Å.

The hydrogen atoms of the host DMB were placed in idealised positions using a riding model, except for the hydroxyl hydrogen atom H3G3, which was assigned from the electron density map and restrained with C-H = 0.84(2) Å to obtain the correct directionality of the hydrogen bond interaction with the O2 oxygen on the adjacent glucose ring. The tertiary and secondary hydrogen atoms were assigned U_{iso} values 1.2 times those of their parent atoms while the methyl hydrogen atoms were assigned U_{iso} values 1.5 times those of their parent atoms. Examination of the electron density map revealed the guest hydrogen atoms, attesting to the high quality of the diffraction data. These were nonetheless placed in idealised positions in a riding model. The hydroxyl hydrogen atoms were placed using the AFIX 147 command which searches for the highest electron density. These refined to reasonable positions consistent with the expected hydrogen bonding scheme. Four water molecules were identified and assigned. The water hydrogen atoms were placed and constrained using the DANG and DFIX commands.

9. Geometrical data defining the conformations of the host molecules

Table S5. Geometrical parameters for the two independent host molecules (A and B) in the crystal of the complex TMA·RSV·6.25H_2O.

The key for these parameters is given as a footnote** in Table S5.

Residue	***r*** (Å)	***D*** (Å)	***a*** (°)	***d*** (°)	***φ*** (°)	D_3 (Å)	***α*** (Å)*	τ_1 (°)	τ_2 (°)
A1	4.513	4.032	114.78	-2.17	118.3(4)	3.154	-0.0515	14.90(13)	3.24(3)
A2	4.485	4.502	115.59	-0.00	119.1(4)	3.287	0.0308	19.34(13)	5.83(4)
A3	3.929	4.395	128.97	2.83	118.7(4)	3.493	0.0248	12.77(13)	5.29(5)
A4	4.498	4.058	115.67	-2.80	117.4(4)	3.222	-0.0593	16.84(13)	3.66(3)
A5	4.503	4.469	114.55	0.70	119.4(3)	3.283	0.0983	19.63(12)	6.44(5)
A6	3.893	4.400	130.35	2.10	118.0(4)	3.473	0.0169	11.68(12)	5.12(6)
*Average esd = 0.0027 Å									
B1	4.609	4.360	112.06	11.45	118.9(4)	3.223	-0.1946	6.98(14)	9.04(7)
B2	4.251	4.386	121.24	-7.20	118.9(4)	3.591	0.2032	23.24(14)	6.22(6)
B3	4.111	4.295	126.93	-4.35	116.4(5)	3.387	-0.0158	27.21(16)	10.31(9)
B4	4.642	4.320	110.44	10.65	119.5(5)	3.229	-0.1837	5.73(17)	7.49(8)
B5	4.220	4.448	122.95	-6.35	118.3(5)	3.631	0.1941	25.34(15)	5.72(4)
B6	4.164	4.265	124.85	-5.35	116.9(5)	3.332	-0.0032	18.50(26)	9.68(8)
*Average esd = 0.0033 Å									

**These parameters are defined as follows:
r, the distance of each O4 atom from the centroid of the O4-polygon;
D, the glycosidic O4···O4′ distance;
a, the O4···O4′···O4″ angle;
d, the O4···O4′···O4″···O4‴ torsion angle;
ϕ, the intersaccharidic angle C1(n+1)-O4(n)-C4(n);
D_3, the O2···O3′ intra-ring distance;
α, the deviation of each O4 atom from the mean O4-plane;
τ_1, tilt angle: the angle between the plane containing the six atoms of a glucopyranose ring (C1, C2, C3, C4, C5, O5) and the mean O4-plane;
τ_2, tilt angle: the angle between the plane containing the atoms O4, C4, C1 and O4′ of a given glucose ring and the mean O4-plane.

Table S6. Geometrical parameters for the host molecule in the crystal of the complex TMB·RSV·5.6H_2O.

Residue	***r*** (Å)	***D*** (Å)	***a*** (°)	***d*** (°)	***φ*** (°)	D_3 (Å)	***α*** (Å)	τ_1 (°)	τ_2 (°)
G1	4.790	4.461	134.3	-12.33	116.5(2)	3.385	0.082(1)	32.37(7)	37.9(1)
G2	5.045	4.483	124.0	22.32	118.5(2)	3.300	0.626(1)	9.71(7)	11.1(1)
G3	5.178	4.412	126.4	9.09	116.5(2)	3.465	-0.330(1)	19.94(5)	20.1(2)
G4	5.145	4.475	119.8	-44.02	118.2(2)	3.473	-0.586(1)	44.08(5)	46.4(1)
G5	4.617	4.313	134.8	23.88	114.9(2)	3.560	0.735(1)	33.26(7)	44.2(1)
G6	5.139	4.350	123.1	19.16	118.3(2)	4.097	0.183(1)	15.40(6)	19.3(1)
G7	5.169	4.387	121.9	-21.35	117.5(2)	3.889	-0.709(1)	49.81(7)	47.5(1)

Table S7. Geometrical parameters for the host molecule in the crystal of the complex DMB·RSV·4.0H_2O.

Residue	***r*** **(Å)**	***D*** **(Å)**	***a*** **(°)**	***d*** **(°)**	***φ*** **(°)**	D_3 **(Å)**	***α*** **(Å)**	$τ_1$ **(°)**	$τ_2$ **(°)**
G1	4.974	4.439	129.50	13.65	117.7	2.796	-0.2874	6.27	8.92
G2	5.151	4.306	125.36	0.84	119.2	2.894	0.1558	9.25	9.49
G3	5.031	4.412	128.62	-13.45	117.8	2.909	0.1876	23.93	24.89
G4	4.920	4.403	130.69	7.47	117.7	2.788	-0.2463	11.64	15.42
G5	5.103	4.312	125.87	5.16	120.0	2.822	-0.0446	2.34	2.98
G6	5.038	4.333	129.33	-3.14	117.5	2.734	0.2099	10.72	12.26
G7	4.993	4.404	128.49	-10.96	118.1	2.877	0.0251	20.58	22.22

10. Experimental and simulated PXRD patterns for the inclusion complexes

The experimental PXRD patterns were collected at 21 °C on a BRUKER D8 Advance X-ray diffractometer using CuK_{α}-radiation (λ = 1.5406 Å) with generator settings 30 kV, 40 mA. Samples were placed on a zero-background sample holder and scanned over the range 4°-40° 2θ with a step-size of 0.02°. Simulated PXRD patterns were computed using the program Lazy Pulverix[1] with the refined single crystal X-ray structural data determined at 173(2) K as input.

In comparing the experimental and simulated patterns, allowance should be made for the distinctly different temperatures involved. This has the general effect that the 2θ-angles of peaks in the calculated patterns (lower temperature) generally tend to occur at higher values than those of the corresponding peaks in the experimental traces. Discrepancies in peak intensities are attributed to preferred orientation in the experimental samples.

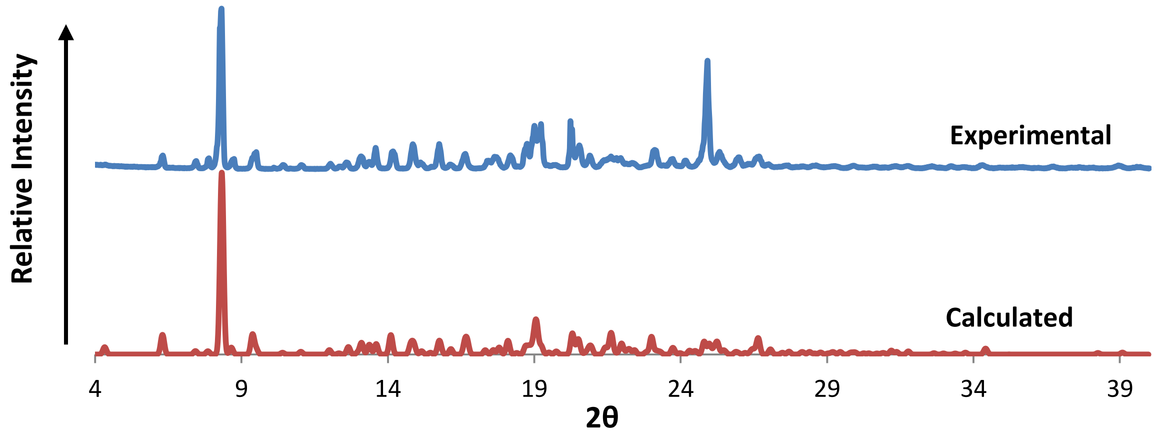

Figure S10. Experimental and calculated PXRD patterns for TMA·RSV·6.25H_2O.

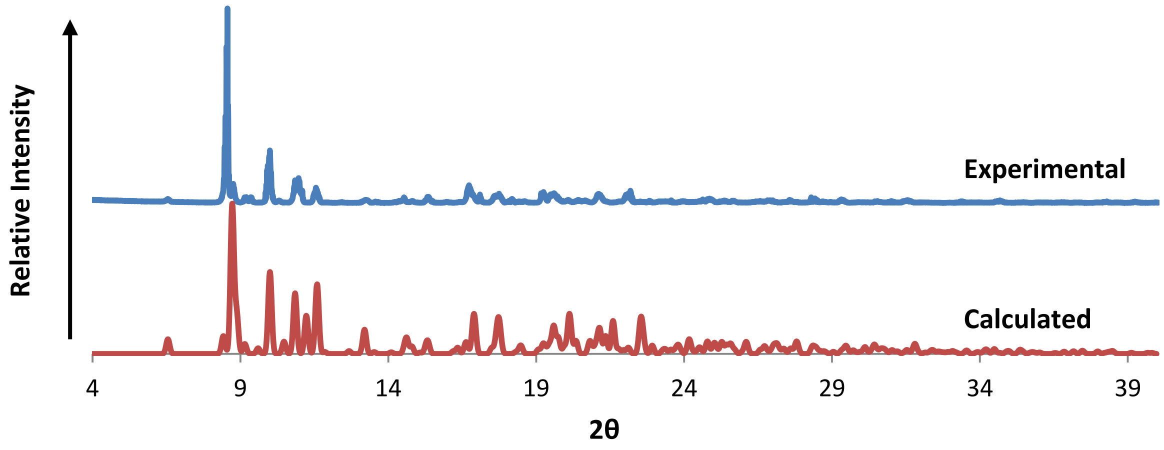

Figure S11. Experimental and calculated PXRD patterns for TMB·RSV·5.6H_2O.

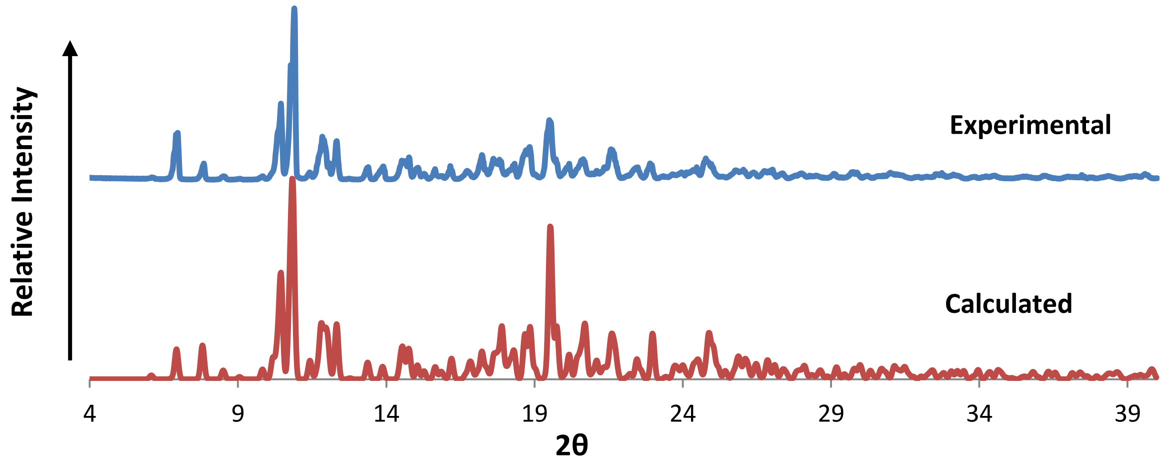

Figure S12. Experimental and calculated PXRD patterns for DMB·RSV·4.0H_2O.

Reference

1. Yvon, K.; Jeitschko, W.; Parthé, E. *LAZY PULVERIX,* a computer program, for calculating X-ray and neutron diffraction powder patterns. *J. Appl. Crystallogr.*, **1977**, *10*, 73-74.
